# Novel Perspectives on the Development of the Amygdala in Rodents

**DOI:** 10.3389/fnana.2021.786679

**Published:** 2021-12-09

**Authors:** Tania Aerts, Eve Seuntjens

**Affiliations:** Laboratory of Developmental Neurobiology, Department of Biology, KU Leuven, Leuven, Belgium

**Keywords:** amygdala ontogeny, brain development, neuronal migration, lineage tracing, molecular patterning, social brain, limbic system

## Abstract

The amygdala is a hyperspecialized brain region composed of strongly inter- and intraconnected nuclei involved in emotional learning and behavior. The cellular heterogeneity of the amygdalar nuclei has complicated straightforward conclusions on their developmental origin, and even resulted in contradictory data. Recently, the concentric ring theory of the pallium and the radial histogenetic model of the pallial amygdala have cleared up several uncertainties that plagued previous models of amygdalar development. Here, we provide an extensive overview on the developmental origin of the nuclei of the amygdaloid complex. Starting from older gene expression data, transplantation and lineage tracing studies, we systematically summarize and reinterpret previous findings in light of the novel perspectives on amygdalar development. In addition, migratory routes that these cells take on their way to the amygdala are explored, and known transcription factors and guidance cues that seemingly drive these cells toward the amygdala are emphasized. We propose some future directions for research on amygdalar development and highlight that a better understanding of its development could prove critical for the treatment of several neurodevelopmental and neuropsychiatric disorders.

## Introduction

**FIGURE 1:**
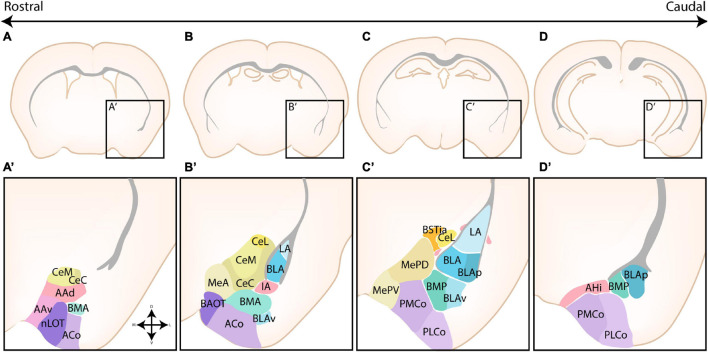
The nuclear subdivisions of the amygdaloid complex. An overview of the nuclei of the basolateral complex (blue/green), the centromedial amygdala (yellow/orange), the cortical nuclei (purple) and transition areas (pink). **(A,A’)** Rostrally located nuclei of the amygdaloid complex include the anterior amygdala area (AAA), further subdivided into a ventral (AAv) and dorsal (AAd) subnucleus, the medial (CeM) and capsular (CeC) subnuclei of the central amygdala (CA), the anterior part of the basomedial nucleus (BMA), the nucleus of the lateral olfactory tract (nLOT) and the anterior cortical nucleus (ACo). **(B,C,B’,C’)** At intermediate levels, the basolateral complex (BLC) is fully visible, encompassing the lateral amygdala (LA), the basolateral amygdala [BLA, including its posterior (BLAp) and ventral (BLAv) divisions] and the anterior (BMA) subnuclei of the basomedial amygdala. The BLC is surrounded by the paracapsular (pink droplets) and main (IA) intercalated cell masses. Along the rostrocaudal axis the lateral (CeL) subnuclei of the CA, the anterior (MeA), posterodorsal (MePD) and posteroventral (MePV) subnuclei of the medial amygdala (MA) and the posteromedial (PMCo) and posterolateral (PLCo) subnuclei of the posterior cortical nuclei (PCo) emerge. The intraamygdaloid part of the bed nucleus of the stria terminalis (BSTia) closely surrounds the MePD and CeL. **(D,D’)** At the caudal pole of the amygdaloid complex, the amygdalo-hippocampal interface (AHi) emerges, joined at this level by the more posterior subnuclei of the BLC [posterior basomedial nucleus (BMP) and BLAp] and PCo (PMCo and PLCo). Figure is based on images from the Allen Brain Atlas (Allen Institute For Brain Science, 2011).

The amygdaloid complex is a cluster of approximately 13 nuclei that are located in the ventro-caudal telencephalon and are considered a part of the limbic system. As a direct consequence of the enormous cellular heterogeneity of the amygdalar nuclei, the optimal classification and the inclusion of certain “transition” structures is heavily disputed (Alheid et al., 1995; Mcdonald, 2006). A first classification divided the amygdalar nuclei into two broad groups based on their functional connectivity with other cortical or subcortical structures and whether their neurons are primarily excitatory or inhibitory. Indeed, as first suggested by Holmgren (1925), the amygdala can be considered an interface structure of pallial and subpallial nuclei. More recent studies have proven that individual nuclei often contain a mixed pallial-subpallial population, additional criteria should therefore be used to properly classify the amygdalar nuclei. On the basis of its cytoarchitecture, neurochemistry and connectivity, the amygdaloid complex was originally subdivided into a basolateral complex (BLC), a centromedial complex and a superficial cortical-like complex (Johnston, 1923; Alheid et al., 1995; McDonald, 1998; Sah et al., 2003). Here, the BLC comprises the lateral amygdala (LA), the basolateral amygdala (BLA), and the basomedial amygdala (BM). The latter two nuclei are sometimes also referred to as the basal amygdala (BA) and the accessory basal amygdala (AB), respectively. The cortical-like nuclei or superficial nuclei include the nucleus of the lateral olfactory tract (nLOT), bed nucleus of the accessory olfactory tract (BAOT) and the cortical nuclei (CoA, anterior and posterior, ACo and PCo, resp). The centromedial nuclei consist of the central amygdala (CA), medial amygdala (MA), and the intra-amygdaloid part of the bed nucleus of the stria terminalis (BSTia). These nuclei are sometimes also referred to as the central or medial extended amygdala if they include the lateral or medial continuum of the BST (BSTL and BSTM), respectively (Alheid and Heimer, 1988; Alheid et al., 1995; Cassell et al., 1999; De Olmos and Heimer, 1999; Alheid, 2003). Three other amygdalar nuclei [the anterior amygdala area (AAA), the amygdalo-hippocampal interface (AHi), and the intercalated cells (ITCs)] do not belong to any of these major groups. We have opted to use this classification due to its widespread use, the (sub)nuclei of the amygdaloid complex are shown in Figure 1. This subdivision somewhat aligns with the notion that the amygdala is a caudal extension of three main structures; the claustrum (basolateral), striatum (centromedial and AAA) and olfactory cortex (cortical-like nuclei) as proposed by Swanson and Petrovich (1998).

Based on the function and connectivity of the individual amygdalar nuclei, a second classification into the frontotemporal (basolateral complex), autonomic (central extended), main olfactory (cortical-like), and accessory olfactory systems (medial extended) is also sometimes used (Swanson and Petrovich, 1998). As evident from this classification, the amygdala could be considered a structure of functionally distinct nuclei that have been clustered as a direct result of their anatomical location. Several arguments that contradict this notion have been raised, including the extensive intraconnectivity of the amygdalar nuclei and the coherent evolution of the amygdaloid complex between several species (Barton et al., 2003).

The basolateral complex is primarily involved in the processing of emotional stimuli, and plays an important role in the generation of conditioned fear responses and anxiety by linking novel stimuli with aversive emotional cues (Swanson and Petrovich, 1998; Baxter and Murray, 2002; Sah et al., 2003; Phelps and LeDoux, 2005; Sah and Westbrook, 2008; Ehrlich et al., 2009; Duvarci and Pare, 2014; Janak and Tye, 2015). The cortical-like nuclei receive direct connections from the main and accessory olfactory bulbs, and are involved in the processing of olfactory stimuli, while the medial amygdala receives direct information from the accessory olfactory bulbs and processes chemosensory and (phero/hor)monal signals to regulate sexual, social, defensive and feeding behaviors *via* modulation of the neuroendocrine system in the hypothalamus (Swanson and Petrovich, 1998; Canteras, 2002; Sah et al., 2003; Keshavarzi et al., 2014). The central amygdala is considered the main output nucleus of the amygdala. It constitutes a major part of “the fear circuit” and can evoke autonomic responses based on perceived emotions (Swanson and Petrovich, 1998; Sah et al., 2003; Phelps and LeDoux, 2005; Sah and Westbrook, 2008; Ehrlich et al., 2009; Duvarci and Pare, 2014; Janak and Tye, 2015; Fadok et al., 2018). In addition to adverse associations, the central amygdala also assigns positive emotions by reinforcing rewarding experiences, best studied in relation to appetite (Baxter and Murray, 2002; Fadok et al., 2018). By integrating emotional cues and sensory input, it serves as an important hub for associative learning. Amygdalar dysfunction lies at the basis of numerous neuropsychiatric and neurodevelopmental disorders, highlighting that precise regulation of the generation, migration and maturation of its cellular components is crucial. Examples include Autism Spectrum Disorder (ASD), anxiety disorders, childhood bipolar, Schizophrenia, Williams Syndrome (WS), Fragile X Syndrome (FXS), mood disorders and Post-Traumatic Stress Disorder (PTSD). As the pathophysiology of the amygdala in neurodevelopmental and neuropsychiatric disorders is not the main focus of this review, we redirect the reader to an excellent review by Schumann et al. (2011).

## Generation of Amygdalar Neurons

### Molecular Patterning of the Developing Telencephalon

Regional patterning is a critical process that occurs during early development, and depends on an interplay between morphogenic factors and gene regulatory networks. Following early patterning events, the embryonic telencephalon gives rise to two main regions; the pallium and the subpallium. Subsequent patterning events further divide the telencephalon in spatiotemporally restricted proliferative regions, which give rise to functionally and morphologically distinct neural subtypes. To better understand amygdalar development, we refer the reader to two excellent reviews on telencephalic patterning (Subramanian et al., 2009; Azzarelli et al., 2015).

**FIGURE 2:**
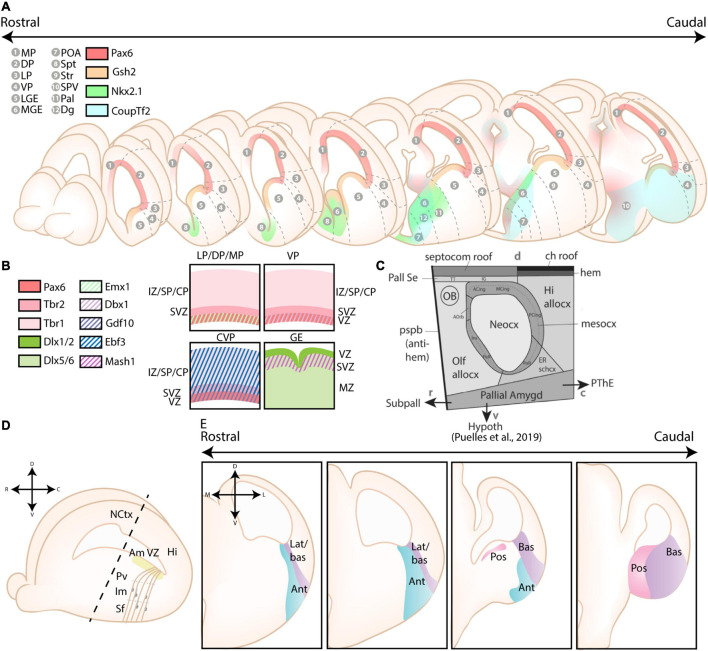
Different models of the pallial cortex and amygdala. **(A)** Overview of common transcription factors and histogenetic regions of the developing murine brain. Transcription factors Pax6, Gsh2, and Nkx2.1 regulate broad telencephalic patterning, and cause the emergence of the pallial, striatal (Str), pallidal (Pal), and diagonal (Dg) sectors. Visible along the rostrocaudal axis are the four regions of the tetrapartite model [Lateral (LP), Ventral (VP), Medial (MP), and Dorsal Pallium (DP)]. Other depicted regions include the lateral, medial and caudal ganglionic eminences (LGE, MGE, CGE, resp.), the preoptic area (POA), the septum (Spt), and the supraopto-paraventricular hypothalamus (SPV). At intermediate and caudal levels, expression of CoupTf2 is shown. **(B)** Schemes depicting the expression of transcription factors in the ventricular zone (VZ), subventricular zone (SVZ) and mantle zone (MZ)/intermediate zone (IZ)/Subplate (SP)/Cortical plate (CP) of the LP/DP/MP, VP, caudoventral pallium (CVP) and ganglionic eminences (GE), highlighting their differences. **(C)** Figure adapted (greyscale) from Puelles et al. (2019), showing a tentative scheme of the concentric ring model of the pallium. **(D)** A side view of the developing murine brain, showing the lateral ventricle at the level of the ventral pallium and amygdalar pallium. Radial glial fibers arising from the ventricular zone of the pallial amygdala (Am VZ, yellow), located at the caudal pole of the telencephalon, course rostroventrally toward the pial surface. The pallial amygdalar nuclei can be divided along the radial axis in periventricular (Pv), intermediate (Im), and superficial (Sf) nuclei. **(E)** Radial view on the pallial amygdalar units along the rostrocaudal axis. The pallial amygdala is divided into a lateral (lat), basal (bas), anterior (ant), posterior (pos) and retroendopiriform (rep, not shown) radial unit. NCtx, NeoCortex; Hi, Hippocampus. Figure is based on images from Garcia-Calero et al. (2020) and Garcia-Calero and Puelles (2021).

The pallium was originally divided into four primary progenitor zones based on the tetrapartite model by Puelles et al. (1999, 2000), including the lateral pallium (LP), the dorsal pallium (DP), the medial pallium (MP), and the ventral pallium (VP) (see Figures 2A,B). This model was widely accepted as the standard model for over 20 years, and only recently challenged by several novel models as critically reviewed in Medina et al. (2021). Relevant for amygdalar development is the concentric ring theory of the pallium (re)introduced by Puelles et al. (2019). This theory includes the allocortical field [olfactory (VP), hippocampal (MP)], and the transitional mesocortical field [cingulate cortex, insular cortex (LP)] as a double ring surrounding the central neocortical island (DP), with separate septal and amygdalar pallial fields located outside this double ring (see Figure 2C; Puelles et al., 2019). A recent paper by Garcia-Calero et al. (2020) then systemically divided the pallial amygdalar components into radial units based on novel gene patterning data and the orientation of radial glia, with each radial unit (lateral, basal, anterior, posterior, and retroendopiriform) containing a periventricular, intermediate, and superficial part (Figures 2D,E). In combination with the concentric ring theory of the pallium, this model elucidated multiple patterning discrepancies that plagued previous models (Puelles et al., 2019; Garcia-Calero et al., 2020). Most notably, the amygdalar radial units lie at 45° relative to the usual coronal section plane, so that the corresponding ventricular zone lies caudal to the amygdala itself and single coronal sections of amygdalar nuclei therefore never revealed their respective progenitor zones. Many older observations and published papers are based on the traditional “coronal” view of the amygdala, implicating a need to reexamine previous findings. Since many of these would require additional analysis, we will focus on earlier conclusions and implement these new insights where possible.

Molecularly, cells located in the ventricular zone (VZ) of the cortex are characterized by their expression of *Emx1* starting from embryonic day (E)9.5, with the exception of the VP (Briata et al., 1996; Gulisano et al., 1996; Yoshida et al., 1997; Puelles et al., 2000; Gorski et al., 2002). Instead, progenitor cells located in the VP express *Dbx1* in their VZ starting from E9.5 (Shoji et al., 1996; Yun et al., 2001; Medina et al., 2004). *Lhx9* was commonly used as a marker for VP-derivatives, however, it was recently clarified that *Lhx9* is specifically expressed in derivatives of the anterior and posterior pallial amygdalar unit, and is completely separated from the VP (Tole et al., 2005; García-López et al., 2008; Puelles et al., 2019; Garcia-Calero et al., 2020; Garcia-Calero and Puelles, 2021). A study by Ruiz-Reig et al. (2018) introduced a novel ventral pallial subdomain, the caudoventral pallium (CVP). This subdomain lacks expression of *Dbx1*, but is characterized by expression of *Gdf10* in its VZ and *Ebf3* in the subventricular/mantle zone (SVZ/MZ) and in CVP-derivatives until P3 (Ruiz-Reig et al., 2018). Contradictory to what its name suggests, this subdomain is part of the pallial amygdalar field and was merely named before the separation of the cortical and amygdalar pallial fields.

The subpallium consists of five major progenitor regions, including the lateral ganglionic eminence (LGE), the medial ganglionic eminence (MGE), the caudal ganglionic eminence (CGE), the preoptic area (POA) and the subpallial septum. In contrast to pallial progenitor cells, subpallial progenitor cells are characterized by their expression of genes from the Dlx-family (Robinson et al., 1991; Anderson et al., 1997; Liu et al., 1997; Puelles et al., 2000) and Mash1 (Guillemot and Joyner, 1993; Casarosa et al., 1999; Marìn et al., 2000; Yun et al., 2002; Long et al., 2009). It is necessary to introduce the ventrally located “pMGE5” region, which was referred to as the anterior entopeduncular area (AEP) in older papers, and recently renamed the caudoventral MGE (cvMGE) or diagonal area (Dg), the latter of which is currently used in the Allen Developing Mouse Brain Atlas (García-López et al., 2008; Bupesh et al., 2011b; Allen Institute For Brain Science, 2013; Puelles et al., 2013, 2016b). The Dg specifically generates *Somatostatin* (*Sst*)-expressing interneurons for the cortex, striatum and amygdala, in contradiction to reports that assigned the most dorsal subdomain of the MGE as the origin of Sst-expressing interneurons (Wonders et al., 2008; Real et al., 2009; Puelles et al., 2016b; Hu et al., 2017).

### Developmental Origin of Amygdalar Neurons

In general the amygdala is an early born structure as its cellular components are largely generated between E10-14 (mouse) (McConnell and Angevine, 1983; Remedios et al., 2007; Cocas et al., 2009; Soma et al., 2009; Vucurovic et al., 2010). Combined data from an older study that injected 3H-Thymidine between E11-E18, and a more recent study that injected BrdU between E10-E14, suggest that cells of the amygdaloid complex are generated following a rostrocaudal gradient (McConnell and Angevine, 1983; Soma et al., 2009). The cells that comprise the centromedial nuclei are generated first around E10, together with layer 1 of the nLOT (McConnell and Angevine, 1983; Remedios et al., 2007; Soma et al., 2009). Injections at E11 label the subdivisions of all amygdalar nuclei appearing in most anterior coronal sections, including layer 2 and 3 of the nLOT, the AAA and anterior parts of the MA, CoA, and BLC, the latter of which is most intensely labeled (McConnell and Angevine, 1983; Remedios et al., 2007; Soma et al., 2009). At E12, neurogenesis of cells that comprise the MA has significantly halted, and labeling shifts more toward the posterior subdivisions of the amygdaloid complex (McConnell and Angevine, 1983; Soma et al., 2009). Some nuclei such as the LA and BM show a simultaneous start of neurogenesis of its anterior and posterior subdivisions (E12), however, the generation of neurons of their respective anterior subdivision either peaks earlier (LA) or their posterior subdivision exhibits prolonged proliferation compared to the anterior subdivision (BM) (McConnell and Angevine, 1983; Cocas et al., 2009; Soma et al., 2009). At E13 the neurogenesis of the ITCs peaks, and labeling of all other nuclei is reduced (McConnell and Angevine, 1983; Cocas et al., 2009; Soma et al., 2009).

While the generation of amygdalar neurons is largely completed by E14, most neural populations have not yet aggregated into definitive deep, intermediate or superficial nuclei, complicating a proper classification of its nuclei at this time point.

#### Basolateral Complex

**FIGURE 3:**
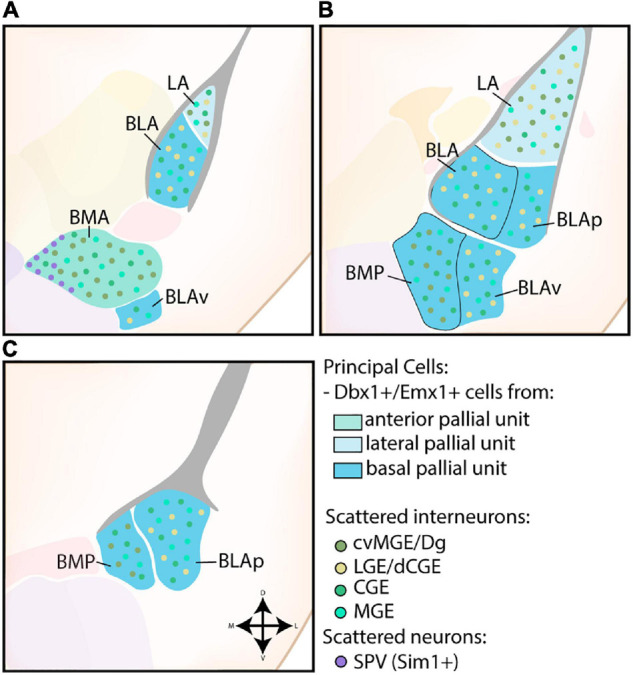
Developmental origin of the basolateral complex. **(A)** The principal cells of the more anterior lateral amygdala (LA) and basolateral amygdala (BLA) are Dbx1-expressing neurons originating in the lateral and basal pallial units The anterior basomedial amygdala (BMA) contains a majority of Dbx1-expressing neurons from the anterior pallial unit, joined by Sim1-expressing neurons from the supraopto-paraventricular hypothalamus (SPV) at its medial side. In addition to these principal excitatory cells, some Emx1-expressing neurons are also present in the LA, BLA, and BMA at this level. The small part of the posterior ventral BLA (BLAv) contains a majority of excitatory Emx1-expressing cells from the basal pallial unit, with some Dbx1-expressing neurons scattered around (not shown). **(B)** The contributions from the Emx1-lineage increase along the rostrocaudal axis. At intermediate levels, more parts of the BLA become visible (BLAp and BLAv), both containing a majority of Emx1-expressing cells from the basal pallial unit, with some Dbx1-expressing cells still present (not shown). **(C)** At caudal levels, only the posterior subdivisions of the BM (BMP) and BLA (BLAp) are visible, both containing a majority of Emx1-expressing neurons with a minority of Dbx1-expressing neurons. Scattered interneurons can be found throughout the entire basolateral complex, with CGE and MGE-derived interneurons inhabiting the entire complex. In contrast, interneurons from the cvMGE/Dg area preferentially inhabit the LA and BM, while only data from the LA and BLA is available for dLGE/dCGE-derived interneurons from the Gsh2-lineage.

The nuclei of the BLC were the first identified amygdalar nuclei and their teardrop morphology, reminiscent of an almond, prompted Burdach (1819) to call this novel area the “amygdala.” The BLC consists of excitatory projection neurons of pallial origin, in combination with scattered inhibitory interneurons from subpallial origin (see Figure 3). As expected from the pallial character of the BLC, analysis of *Pax6*^sey/sey^ mice by Tole et al. (2005) showed *Emx2*-independent misdevelopment of several nuclei of the amygdaloid complex, including the entire BLC. A combination of gene expression analysis, *in utero* gene transfer studies and lineage tracing revealed that the majority of projection neurons in the LA and anterior BLA nuclei originate from a *Dbx1*+ progenitor pool starting from E11.5 (Medina et al., 2004; Bielle et al., 2005; Hirata et al., 2009; Soma et al., 2009; Waclaw et al., 2010; Puelles et al., 2016a). As expected, Tlx loss-of-function (LOF) mice, which completely lose *Dbx1* expression in the telencephalon, exhibit a severe reduction of the LA and BLA (Stenman et al., 2003). In contrast, expression analysis and lineage tracing of *Emx1* has shown that a subset of cells inhabit more posterior regions of the BLC, complementary to the anterior position of *Dbx1*-lineage cells (Puelles et al., 2000; Gorski et al., 2002; Medina et al., 2004; Cocas et al., 2009). These *Dbx1*+ and *Emx1*+ progenitor pools were originally believed to be the cortical VP and cortical LP, respectively, but the amygdalar pallium is now understood to be completely separate from cortical pallial regions (Puelles et al., 2000, 2019; Gorski et al., 2002; Medina et al., 2004; Tole et al., 2005; Cocas et al., 2009; Garcia-Calero et al., 2020). All pallial amygdalar units were described to contain a mixture of *Dbx1*+ and *Emx1*+ cells, however, the rostral parts of the lateral, basal, and anterior amygdalar subdivisions seem to contain a high proportion of *Dbx1*-lineage cells, while the basolateral and posterior amygdalar units contain more *Emx1* expression (Puelles et al., 2016a; Garcia-Calero and Puelles, 2021). A detailed tracing of radial glia scaffolds indicated that the excitatory cells of the lateral and basolateral compartment originate from the ventricular region of the lateral and basal radial unit, respectively (Garcia-Calero et al., 2020).

While these studies mostly indicate a shared molecular origin of projection neurons of the LA and BLA, LOF studies have revealed subtle differences between them. *Gsh2*-conditional LOF (*Foxg1*^tTA/+^), which show an expansion of pallial tissue into the dLGE, predominantly resulted in an increased cell number and size of the LA (Waclaw et al., 2010). A subpopulation of *Gsh2*-lineage cells (LA: 35%, BLA: 11%) within the BLC was found to co-express *Tbr1*, likely as a result of fate switching and cell movements at the Pallial-Subpallial Boundary (PSB) before E15.5 (Cocas et al., 2009). Intriguingly, conditional *Pax6* LOF mice (*Gsh2*-lineage) exhibited a reduction of *Dbx1* expression in the VP and presumable amygdalar pallium and resulted in decreased *Tbr1*-expressing cells in the LA, but not BLA (Cocas et al., 2011). These findings suggest that not all pallial amygdalar units are equally sensitive to disruption of the PSB. While the interplay between *Gsh2* and *Pax6* expression on the PSB has been particularly well-studied, how this translates to the different amygdalar pallial units remains unknown (Stoykova et al., 1996, 2000; Corbin et al., 2000; Toresson et al., 2000; Yun et al., 2001; Carney et al., 2009). It would be interesting to reinvestigate the subtle differences between the LA/BLA and the contribution of excitatory *Gsh2*-lineage in light of the novel radial unit model.

A subset of BM cells are likewise generated from *Dbx1*- and *Emx1*-lineages (Gorski et al., 2002; Cocas et al., 2009; Hirata et al., 2009; Waclaw et al., 2010; Puelles et al., 2016a), as expected from their classification within the anterior (anterior BM, BMA) and basal (posterior BM, BMP) radial units (Garcia-Calero et al., 2020). Surprisingly, Tang et al. (2012) reported that the majority of glutamatergic projection neurons in the BM originate from the CGE around E12.5-14.5. The transcription factor *CoupTf2* plays a critical role in the generation and migration of these cells, as conditional knockout of *CoupTf2* in the ventral telencephalon resulted in a complete ablation of glutamatergic (*Glu2R*+, *Pax6*+) cells in the BM nuclei (Tang et al., 2012). It is worth noting that their glutamatergic character and strong *Pax6* expression indicates a pallial rather than subpallial origin. Questions should have risen from their use of Rx-Cre mice to drive conditional *CoupTf2* deletion in the CGE (Tang et al., 2012). Klimova et al. (2013) showed that gene expression of *CoupTf2* is also deleted in regions of the pallium in this mouse line. This corroborates the now generally accepted presence of pallial tissue surrounding the CGE at the caudal end of the telencephalon (Puelles et al., 2013; Ruiz-Reig et al., 2018). Nonetheless, *Pax6*+ cells in their conditional *CoupTf2* knockout mice did not overlap with the *Lhx2*+ population, a marker that was later found to label cells of the anterior amygdalar unit together with *Lhx9* (Tang et al., 2012; Garcia-Calero and Puelles, 2021). These cells could therefore originate in the basal radial unit, however, more research is required to investigate the relationship between expression of *CoupTf2* and the novel amygdalar units. Notably, *CoupTf2* expression in both pallial and subpallial progenitor zones of the amygdala has been reported in chicken, and in sauropsids two *CoupTf2-*positive pallial divisions have been identified, with strongest expression reported in the caudalmost division, strikingly comparable to the strong *CoupTf2* expression in the more caudal mouse pallial amygdalar units (reviewed in Medina et al., 2021). Peculiarly, a subset of hypothalamus-derived cells (*Sim1*+, *Otp-*), originating in the supraopto-paraventricular hypothalamus (SVP), was found to populate a shell-shaped region surrounding the BMA nucleus at its medial side (Figure 3; García-Moreno et al., 2010; Garcia-Calero et al., 2021; Morales et al., 2021).

Similar to their distribution in the cerebral cortex, small interneurons can be found scattered throughout the BLC (Figure 3). Fate-mapping of *Nkx2.1*-lineage neurons and homotopic transplantation of E13.5 MGE and CGE cells indicates that the majority of scattered interneurons in the BLA/LA originate from the MGE (Nery et al., 2002; Xu et al., 2008). At least a subset of those are generated in the Dg/cvMGE, which were shown to extensively colonize the BLC (Real et al., 2009; Vucurovic et al., 2010; Puelles et al., 2016b). Intriguingly, at E16.5 Real et al. (2009) reported a strong presence of *Sst*-expressing neurons from the cvMGE/Dg within the LA and BLA, with an almost complete absence in the BM, while Puelles et al. (2016b) instead reported numerous early-born *Sst*-expressing cells from the cvMGE/Dg in the LA and BM, with only some scattered cells present in the BLA. This highlights that contradictory reports might result from arbitrary annotations of the amygdalar nuclei, without guidance from markers that delineate them. The *Gsh2*-lineage also contributes interneurons to the BLA and LA, unfortunately the BM was not investigated (Waclaw et al., 2010; Cocas et al., 2011). Since these cells are not observed with *Isl1*-lineage tracing, they likely originate from the dLGE or dCGE (Waclaw et al., 2010). Lineage tracing and studies with grafted E14.5 *5HT3aR*:GFP+ cells from the CGE confirmed that at least a subset of BLA/LA interneurons co-express *Prox1* and originate in the CGE “proper” (Vucurovic et al., 2010; Touzot et al., 2016). Whether they overlap with the *Gsh2*-lineage is currently not known. A transplantation of homotopically grafted CGE cells resulted in colonization of the BM nucleus, but these included pallial glutamatergic neurons, and only 29% of all grafted cells were GABAergic (Nery et al., 2002). *GAD67*+ cells in the BM were not affected in conditional *CoupTf2* LOF mice, further arguing against a CGE/POA origin (Tang et al., 2012).

#### Centromedial Complex/Extended Amygdala

Swanson and Petrovich (1998) postulated that the centromedial nuclei are a ventromedial expansion of the striatum based on the presence of GABA and striatal neuropeptide expression. The subpallial nature of this complex was corroborated by gene expression analysis (*Dlx, Nkx2.1*, *Isl1*, *Lhx6*, *Lhx7/8*, *Emx1*, *Tbr1*, …), LOF studies (*Gsh2*, *Nkx2.1*, *Tlx*, *Pax6*, *Emx2*) and fate-mapping of *Dlx5/6*-lineage cells (Puelles et al., 1999, 2000; Gorski et al., 2002; Stenman et al., 2003; Medina et al., 2004; Tole et al., 2005; Waclaw et al., 2010; Wang et al., 2011).

##### Central Amygdala

**FIGURE 4:**
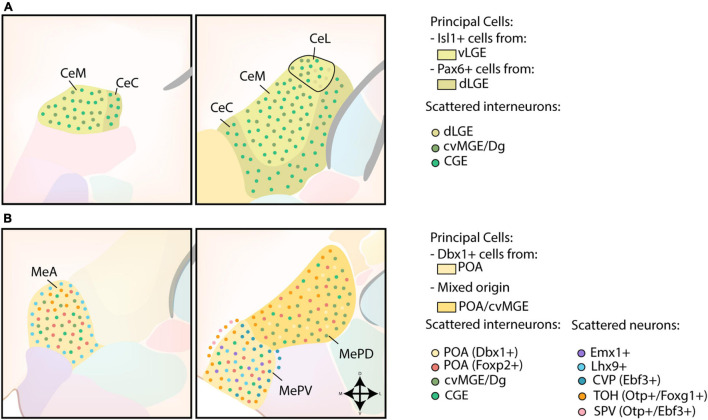
Developmental origin of the centromedial amygdala. **(A)** Schematic overview of the neuronal origin of cells within the central amygdala (CA). Isl1-lineage cells from the ventral LGE (vLGE) inhabit the medial CA (CeM) and the centromedial part of the lateral CA (CeL), while Pax6-expressing cells from the dorsal LGE (dLGE) inhabit the capsular CA (CeC), with the exception of its ventromedial part, and the lateral part of the CeL. Presumptive CGE-derived interneurons can be found throughout the entire CA. Interneurons from the cvMGE instead prefer the CeM and CeL, and seem to avoid the CeC. **(B)** Schematic overview of the neuronal origin of cells within the medial amygdala (MA). POA-derived Dbx1-expressing neurons are spread throughout the entire medial amygdala (MA), but preferentially localize to the anterior MA (MeA) and posteroventral MA (MePV). They are complemented throughout the MA by Foxp2-expressing cells from the POA. Lhx9-expressing neurons, originating in the anterior amygdalar unit, populate a ring-shape surrounding the MeA and the core region of the MePV. Some Emx1-expressing cells were also identified in the MePV. Within the MePV, cells from the caudoventral pallium (CVP) and telencephalon-opto-hypothalamic domain (TOH) occupy specific niches. While cells from the TOH preferentially inhabit the medial side of an imaginary shell surrounding the MePV, cells from the CVP preferentially occupy the dorsolateral edge of the MePV. The TOH-derived cells are also present in the superficial layer of the MA, together with Otp+/Ebf3+ cells from other domains of the supraopto-paraventricular hypothalamus (SPV). TOH-derived neurons are also present within the posterodorsal MA (MePD), where they prefer to inhabit its medial part. A population of CGE-derived interneurons is presumed to populate the MA. In addition, small interneurons from the cvMGE/Dg are scattered within the MeA and the MePD.

The strong expression of *Dlx5*, weak expression of *Nkx2.1*, *Lhx6*, *Lhx7/8* and absence of pallial markers *Tbr1* and *Emx1* in the CA further indicates a striatal character (Puelles et al., 1999, 2000; Gorski et al., 2002; Medina et al., 2004; García-López et al., 2008). A schematic overview of the developmental origin of cells within the CA is shown in Figure 4A. Gene expression analysis and fate-mapping of *Isl1*-lineage cells proved that the principal cells in the CA originate from the ventral LGE (vLGE), and show medium spiny-like morphologies (Waclaw et al., 2010; Bupesh et al., 2011a). *Via* lineage tracing and cell tracking components, vLGE-derived cells were found to preferably inhabit the medial CA (CeM) and the centromedial part of the lateral CA (CeL), with an almost complete absence in the capsular CA (CeC) (Waclaw et al., 2010; Bupesh et al., 2011a). In contrast, *Pax6*-expressing cells from the dorsal LGE (dLGE) seem to inhabit complementary regions of the CA; the CeC contains abundant dLGE-derived cells, the lateral part of the CeL shows a moderate amount while the CeM completely lacks these cells (Puelles et al., 2000; Bupesh et al., 2011a). Surprisingly, while *Gsh2* LOF severely affects the size of the vLGE and the development of the striatum, the size of the CA was mostly unaffected (Toresson and Campbell, 2001; Stenman et al., 2003; Waclaw et al., 2010). This finding prompted Waclaw et al. (2010) to hypothesize that vLGE-derived CA neurons arise from its most ventral portion, which is relatively unaffected in Gsh2 mutants.

The CeM and CeL also contain an important population of cvMGE-derived cells, generated starting from E10.5 (García-López et al., 2008; Bupesh et al., 2011a; Puelles et al., 2016b). An earlier study by Nery et al. (2002) concluded that a subset of CA cells is derived from the CGE, based on homotypic transplantation studies. However, some caution is advised since the transplanted cells could include the Dg/cvMGE. Another study that tried to decipher the origin of several amygdaloid nuclei by *in utero* gene transfer of eGFP described labeled cells that co-expressed Dlx5 in the CA when the CGE was targeted (Soma et al., 2009). As the cvMGE was separately investigated in this study, these cells must originate in the CGE “proper” (Soma et al., 2009). The authors themselves conclude that the CA might partially be derived from the dLGE or dCGE. Similar to the BM, a shell of hypothalamic-derived cells (*Sim1*+, *Otp-*) was reported to surround the CA (García-Moreno et al., 2010), however, this finding has not been replicated in any of the *Otp/Sim1* reporter mice (Garcia-Calero et al., 2021; Morales et al., 2021).

##### Medial Amygdala

The medial amygdala (MA) seems to have a different developmental origin, and should rather be considered a ventromedial extension of the pallidum as postulated by Puelles et al. (1999). The developmental origin of MA neurons is schematically presented in Figure 4B. *Shh*- and *Dbx1*-lineage tracing experiments revealed a high density of labeled cells in the anterior MA (MeA) and posteroventral MA (MePV), with a smaller amount located in the posterodorsal MA (MePD) (García-López et al., 2008; Hirata et al., 2009; Carney et al., 2010; Waclaw et al., 2010; Kanatani et al., 2015; Puelles et al., 2016a). In contrast to the principal projection neurons of the BLC, these *Dbx1*-expressing cells are generated in the POA at an earlier timepoint (E9.5-11.5) and are inhibitory (GABAergic) in nature (García-López et al., 2008; Hirata et al., 2009; Waclaw et al., 2010; Kanatani et al., 2015; Puelles et al., 2016a). A second subpopulation of POA-derived GABAergic neurons destined for the MA was recently identified (Lischinsky et al., 2017). These cells are characterized by their expression of *Foxp2*, and are complementary to the *Dbx1*-lineage cells within the MA (Lischinsky et al., 2017). Intriguingly, these two POA-derived subpopulations might contribute to the sexual dimorphism of the MA. Indeed, postnatally these populations differ in their expression of steroid pathway proteins between sexes and are activated in a sex-specific manner during mating (Lischinsky et al., 2017).

In addition to the POA, the cvMGE was identified as a potential source of interneurons in the MeA and MePD. However, the presence of cvMGE/Dg-derived neurons in the MA remains controversial, since only few *Sst-*expressing interneurons are shown to invade the MA (Real et al., 2009; Puelles et al., 2016b). Instead, expression of *Calbindin* was previously used to link these cells to the cvMGE (García-López et al., 2008; Bupesh et al., 2011b). Observations from homotopic transplantation experiments made by Nery et al. (2002) indicate that at least some MA neurons originate in the CGE, although these could also include the cvMGE. While Vucurovic et al. (2010) found no *5HT3aR*:GFP+ grafted cells (E14-E14.5) from the CGE, Touzot et al. (2016) identified some *5HT3aR*:GFP+ cells within the MA, of which 53% co-expressed *Prox1* (CGE). As expected from the major subpallial contributions to this nucleus, it was not affected in *Pax6*^sey/sey^ mice (Tole et al., 2005).

Excitatory cells of the MA are similarly diverse, with origins ranging from telencephalic to hypothalamic progenitor pools. Fate-mapping of *Emx1*-lineage cells showed an absence in the MePD, although some *Emx1*-expressing cells were identified in the MePV (Gorski et al., 2002). A subset of Dbx1-lineage neurons in the MeA and MePV express *Lhx9* and are hypothesized to be tangentially migrated cells from the anterior radial amygdala unit (García-López et al., 2008; Bupesh et al., 2011b; Puelles et al., 2016a; Garcia-Calero et al., 2020; Garcia-Calero and Puelles, 2021). Within the MeA, the *Lhx9*-expressing cells preferentially colonized the shell and avoided the core subdivision, while *Lhx9*-expressing cells in the MePV preferentially inhabited the core and avoided the shell (Garcia-Calero and Puelles, 2021). These neurons might overlap with a *Tbr1*-expressing population identified in the ventral-most portion of the MePV by Carney et al. (2010).

*Ebf3/Tbr1* co-expressing glutamatergic cells originating in the CVP were reported to form a shell surrounding the MePV, thereby separating it from the BM laterally and from the MePD dorsally (Ruiz-Reig et al., 2018). These CVP-derived cells perfectly complement the abundant *Lhx9* cells in the core of the MePV (Ruiz-Reig et al., 2018; Garcia-Calero and Puelles, 2021). While our current understanding is that the cortical and amygdalar pallial fields should be completely separated (Puelles et al., 2019), and thus the description of the CVP as a cortical pallial area is inherently wrong, these authors likely identified a *Dbx1*-negative pallial amygdalar progenitor zone that delivers cells to the MePV. Bupesh et al. (2011b) likely already identified these cells in 2011, as he observed labeled cells in the MePV when a cell tracker (CMFDA) was placed in the “caudal ventral pallium.” Targeting the more “caudal ventral pallium” resulted in labeled cells located superficially around the MePV, while placing CMFDA in more rostral parts of the “ventral pallium” resulted in labeled cells in the MeA, which supports that the cell tracker was placed in the more rostral progenitor zone of the anterior amygdala unit in the latter case (Bupesh et al., 2011b). As expected from its presumed pallial origin, *Ebf3*-expressing cells are strongly reduced in *Pax6*KO embryos (Ruiz-Reig et al., 2018).

The VZ of the SPV generates *Otp*-expressing cells starting from E11, which were found to cross the telencephalic-hypothalamic border toward the MA (García-Moreno et al., 2010; Bupesh et al., 2011b; Morales et al., 2021). Ninety-nine percent of all *Otp*-expressing cells in the medial amygdala co-expressed *Foxg1* and were generated in the telencephalon-opto-hypothalamic domain (TOH), a dorsal subdomain of the SPV (Morales et al., 2021). *Otp/Foxg1* co-expressing cells preferentially inhabited the anterodorsal part of the MeA, the medial part of the MePD and a ring surrounding the MePV, thereby agglomerating at its medial edge (Morales et al., 2021). An additional *Otp*+ cell population, located between the MePV and the superficial layer of the medial nucleus, was likely generated in the more central or ventral parts of the SPV based on their co-expression of *Ebf3* and lack of *Foxg1* expression, although confirmation of this birthplace is necessary (Ruiz-Reig et al., 2018). The significant contribution of *Otp*-derived cells to the MA was corroborated by the analysis of *Otp*^–/^*^–^* mice, in which the size of the MA nucleus was severely reduced (García-Moreno et al., 2010). By analyzing the gene expression of *Pax6*, Bupesh et al. (2011a) identified an additional migratory stream that seemingly originated in the prethalamic eminence (PTE) and was bound for the MA. However, the hypothalamo-amygdalar corridor (HyA) lies rostrolaterally from the PTE, in close relation to it (Garcia-Calero et al., 2021). Since no other gene expression markers were used except for *Pax6*, we cannot exclude that the observed cells do not originate from other sources. Puelles et al. (2016a) similarly observed *Dbx1*-expressing cells originating from the SPV/PTE-region, but noted that distinguishing between both regions proved difficult. Lineage tracing and/or cell tracking experiments could help further prove this alternative hypothalamic origin.

##### Bed Nucleus of the Stria Terminalis

**FIGURE 5:**
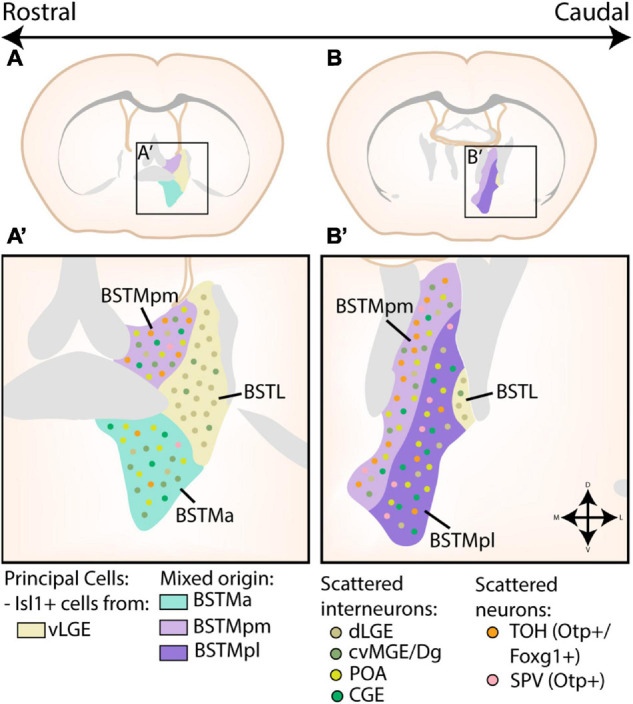
Developmental origin of the Bed Nucleus of the Stria Terminalis. **(A,A’)** At rostral levels, the medial part of the bed nucleus of the stria terminalis (BSTM) contains a posteromedial (BSTMpm) and anterior (BSTMa) part. The entire BSTM contains inhibitory neurons derived from the caudoventral MGE or Diagonal area (cvMGE/Dg), CGE and preoptic area (POA). In addition, the BSTMpm contains a majority of telencephalon-opto-hypothalamic domain (TOH)-derived cells, although some cells originating from other supraopto-paraventricular (SPV) domains are also present. A small minority of TOH/SPV cells can also be found in the BSTMa. The lateral part of the bed nucleus of the stria terminalis (BSTL) is also visible at this level. The principal cells of this subnucleus are Isl1+ cells originating in the ventral LGE (vLGE), which are joined by some Pax6+ cells from the dLGE. Some of these Pax6+ cells were also found to invade the BSTM, while a minority of cvMGE-derived cells from the BSTM also inhabit the BSTL. **(B,B’)** At more caudal levels, the BSTM is divided into a posteromedial (BSTMpm) and posterolateral (BSTMpl) part. Here, the BSTMpm again contains the majority of TOH/SPV-derived cells, especially in its medial part, although the BSTMpl also contains scattered TOH/SPV-derived cells. The distribution of cvMGE/CGE/POA/dLGE/vLGE-derived cells in both the BSTM and BSTL remain similar to those at more rostral levels. Panels **(A,B)** are based on images from the Allen Brain Atlas, with the different subnuclei of the BST translated to extended amygdala terms *via* the scheme of Dong et al. (2001) and Allen Institute For Brain Science (2011).

The BST was originally subdivided into the BSTL and BSTM based on the similarity of its neurochemistry to other nuclei of the centromedial complex (Alheid and Heimer, 1988; Alheid et al., 1995; Cassell et al., 1999; De Olmos and Heimer, 1999; Alheid, 2003). The BSTia is often considered a caudal extension of the BSTM (García-López et al., 2008). The BST was later redivided into approximately 20 subgroups; an excellent overview scheme of different models of BST parcellation can be found in Dong et al. (2001). In general, the BST is marked by the expression of *Nkx2.1* and *Lhx6* (Puelles et al., 2000; García-López et al., 2008; Xu et al., 2008). The developmental origin of BST neurons is schematically shown in Figure 5.

Based on cell tracking (CMFDA) and gene expression studies, the BSTL was found to contain a mixture of vLGE-derived *Isl1*-expressing neurons and dLGE-derived *Pax6*-expressing neurons, although this latter cell population was less significant than in the CA (Bupesh et al., 2011a; Tinterri et al., 2018). In contrast to the vLGE-derived neurons of the CA, BSTL *Isl1*-expressing neurons are derivatives of corridor cells as evident from their migratory route and dependence on *Ebf1* expression (Tinterri et al., 2018). A small amount of cvMGE-derived interneurons are also present in the BSTL, which could explain the expression of *Nkx2.1* and *Lhx6* in this region (García-López et al., 2008; Bupesh et al., 2011a; Puelles et al., 2016b). García-López et al. (2008) had previously already suggested a partial striatal origin for the BSTL, based on the weak expression of *Nkx2.1* and strong expression of *Dlx5* and *Lmo4* at later developmental stages.

The BSTM can be further subdivided into an anterior (BTSMa), posteromedial (BSTMpm), and posterolateral (BSTMpl) part. In-depth analysis of *Sst*-expressing neurons in combination with cell tracking studies and gene expression analysis indicates that a significant portion of the BSTMa, BSTMpm, and BSTia is populated by cvMGE-derived neurons (García-López et al., 2008; Real et al., 2009; Bupesh et al., 2011b; Puelles et al., 2016b). In addition, transplantation experiments of presumed CGE-derived cells identified labeled cells within BST (Nery et al., 2002; Vucurovic et al., 2010). Intriguingly, the dLGE also contributed a marginal amount of cells to the BSTM (Bupesh et al., 2011a). Moreover, when a cell tracker was placed in the POA, labeled cells that co-expressed *Shh* were identified in the BSTM (García-López et al., 2008; Bupesh et al., 2011b). These cells are complemented with cells from the TOH, which preferentially colonize the BSTMpm, although scattered cells are also present in the BSTMpl while the BSTMa only contains a minority of TOH-derived cells (García-Moreno et al., 2010; Bupesh et al., 2011b; Morales et al., 2021).

#### Cortical-Like Nuclei

**FIGURE 6:**
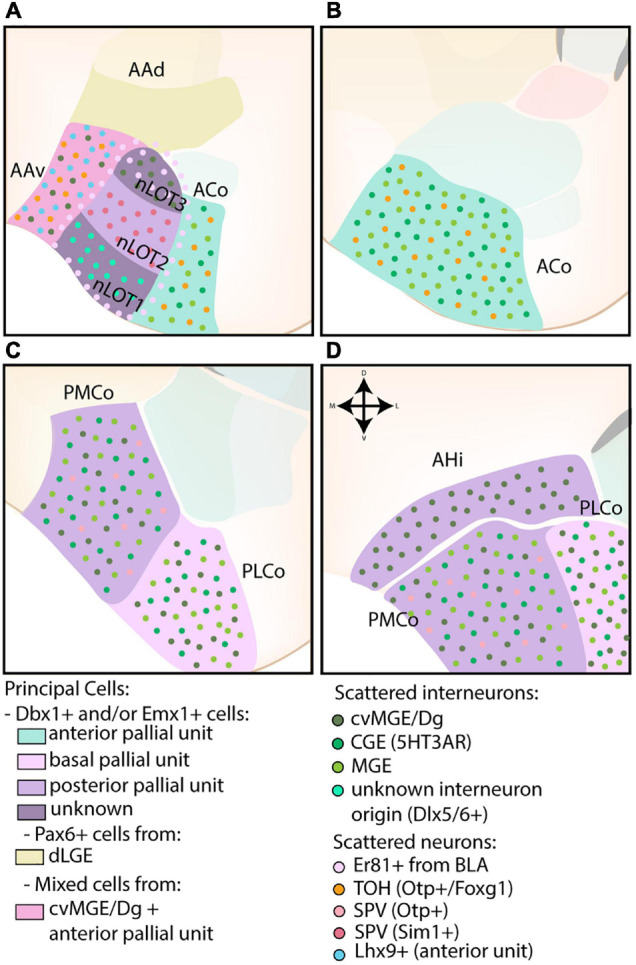
The developmental origins of the corticoamygdaloid and transition nuclei. **(A)** The nucleus of the lateral olfactory tract (nLOT), located at more rostral levels, contains three layers that originate from distinct progenitor pools. While the exact molecular origin of layer 1 and 3 (nLOT1/3) is currently unknown, nLOT3 was found to contain an abundance of caudoventral MGE/Diagonal area (cvMG/Dg)-derived cells and Er81-expressing cells, the latter that might originate from the basolateral amygdala (BLA, basal pallial unit) and surrounds the entire nLOT. Scattered interneurons of unknown origin (Dlx5/6) are present in the nLOT1, which might also express Lhx9 (controversial). In contrast to nLOT1/3, the origin of nLOT2 is well-known and its principal cells are derived from the posterior pallial unit. Emx1+ cells are complemented by Sim1+ cells from the supraopto-paraventricular hypothalamus (SPV). The dorsal part of the anterior amygdala area (AAd), also visible at this level, contains a significant proportion of dLGE-derived Pax6-expressing cells. In contrast, the ventral part (AAv) contains a mix of cvMGE-derived cells, Lhx9+ cells from the anterior amygdala unit and some Otp+/Foxg1+ cells from the hypothalamus (TOH). **(A–D)** The corticoid nuclei of the amygdala are divided along the rostrocaudal axis into an anterior (ACo), posteromedial (PMCo), and posterolateral (PLCo) part. They are all derived from different pallial amygdalar units (anterior, posterior, and basal, resp.) and contain a mix of Dbx1+ and Emx1+ cells. Both the ACo and PMCo contain cells from hypothalamic origin (SPV), with those from ACo well-annotated to the TOH, and those from PMCo currently unknown. The entire corticoid nuclei contain scattered MGE- and CGE-derived interneurons, with the PMCo and PLCo additionally containing a subset of cvMGE/Dg-derived interneurons. **(D)** The amygdalo-hippocampal interface (AHi) is visible at the most caudal level and consists of cells from the posterior pallial amygdala unit (mostly Emx1+) and cvMGE-derived interneurons.

These superficial structures have a trilayered morphology and are sometimes considered extensions of the piriform cortex (Alheid et al., 1995; Sah et al., 2003; Mcdonald, 2006). Consequently, the cortical-like nuclei of the amygdala predominantly express pallial markers (Puelles et al., 2000; Gorski et al., 2002; Medina et al., 2004; Tole et al., 2005; Remedios et al., 2007; García-López et al., 2008; Hirata et al., 2009). The developmental origin of the cortical-like nuclei are shown in Figure 6.

##### Nucleus of the Lateral Olfactory Tract

The laminated structure of the nLOT resembles the pallium, yet its transcriptional signatures indicate that cells within the three layers originate from distinct embryonic progenitor regions. The developmental origin of the nLOT is represented in Figure 6A. In particular, only layer 2 (nLOT2) contains cells derived from the *Emx1*-lineage (Gorski et al., 2002), while lineage tracing studies of *Dbx1-*lineage mark cells in both nLOT1 and nLOT3 (Puelles et al., 2016a). Functional analysis clarified that both nLOT2/nLOT3 require *Pax6* for correct development, as these layers are completely absent in *Pax6*^sey/sey^ mice, while nLOT1 was correctly formed (Tole et al., 2005). The dependence of nLOT2/3 development on Pax6 expression is reflected by expression of its downstream targets that mark this region such as *Tbr1*, *Ngn2*, and *SCIP* (Puelles et al., 2000; Medina et al., 2004; Remedios, 2004; Remedios et al., 2007; Soma et al., 2009). Cells destined for the nLOT1 are generated at an earlier timepoint (E10.5) as compared to nLOT2/3 (E11.5) (Remedios et al., 2007). While earlier publications described a strong expression of *Lhx2* and *Lhx9* in nLOT1 (Remedios, 2004; Tole et al., 2005; García-López et al., 2008), a more recent publication taking into account the novel radial dimension of the amygdala describes the whole nLOT as *Lhx9*-negative (Garcia-Calero and Puelles, 2021). Without the use of other markers, it is not clear whether the positive region below the nLOT belongs to the nLOT1 or the AAA. The lack of *Lhx9*-expressing cells in nLOT3 indicates a lateral or basal pallial amygdalar origin (García-López et al., 2008; Puelles et al., 2016a; Garcia-Calero and Puelles, 2021). Based on expression of *Er81*, a stream of cells seems to emerge from the horn of the medial BLA toward the nLOT, thereby surrounding the whole nLOT and specifically invading nLOT3, however, more research is required to confirm this observation (Garcia-Calero et al., 2020). The presence of Dg/cvMGE-derived interneurons in the nLOT3 is evident from analysis of *Sst* and *Lhx6* (García-López et al., 2008; Real et al., 2009; Puelles et al., 2016b).

The developmental origin of nLOT2 is best characterized, and was described to reside in a unique division of the dorsal pallium around E11.5-12.5 (Remedios et al., 2007). This progenitor region, referred to as the caudoventral dorsal pallium (cvDP), was thought to be located in the caudal telencephalon, ventrally abutting the lateral ventricle in between the MP and VP/LP (Remedios et al., 2007). The DP character of this progenitor region was proposed based on its expression of *SCIP*, *Emx1*, and *Tbr1*, and absence of *Cad8*, *Sfrp2*, and *Wnt2b*, markers of the LP, VP, and MP, respectively (Medina et al., 2004; Tole et al., 2005; Remedios et al., 2007; Soma et al., 2009). The complete absence of nLOT2 in mice models that show extensive disruption of cortical development (*Lhx2* and *Tbr1* single LOF mutants, and *Emx1/Emx2* double LOF mutants) further seemed to support a cortical origin (Remedios, 2004; Remedios et al., 2007). However, the reintroduction of the concentric ring theory of the pallium rendered the previous name (cvDP) unfit for an amygdalar progenitor region (Puelles et al., 2019). In a later publication these cells were shown to originate in the now called “posterior amygdalar radial unit,” or more specifically at the rostromedial subdivision of the amygdala-hippocampus interface (AHi) (Garcia-Calero et al., 2021). Of note, both sets of authors (Remedios et al., 2007; Garcia-Calero et al., 2021) seem to describe the same region, and merely disagree on the terminology. These telencephalic nLOT2 cells were found to be complemented by cells from the SPV, which enter the telencephalon *via* the hypothalamo-amygdalar corridor (HyA, see below), precisely at the posterior amygdalar progenitor region where the other nLOT2 cells are generated (Garcia-Calero et al., 2021). nLOT2 lacks expression of *Lhx6* and *Gad1*, and seems devoid of *Dbx1-* and *Dlx5/6*-derived neurons (Remedios et al., 2007; Puelles et al., 2016a).

##### Bed Nucleus of the Accessory Olfactory Tract

The developmental origin of the BAOT remains largely obscure to this day. The BAOT is characterized by expression of *Lhx9*, *Tbr1*, and contains fate-mapped *Emx1*-lineage cells (Gorski et al., 2002; Medina et al., 2004; García-López et al., 2008; Garcia-Calero and Puelles, 2021). While the BAOT was previously hypothesized to be of VP origin based on its expression of *Lhx9*, the now corresponding anterior radial unit does not match molecularly with the BAOT (Garcia-Calero et al., 2020; Garcia-Calero and Puelles, 2021). The BAOT is therefore currently hypothesized to be of posterior amygdalar or PTE origin, two regions that are also marked by *Lhx9* and *Tbr1* expression (Garcia-Calero and Puelles, 2021).

##### Cortical Nuclei

The cortical nuclei (CoA) of the amygdaloid complex comprise the ACo and the PCo, the latter of which contains a posterolateral (PLCo) and posteromedial (PMCo) subdivision. The developmental origin of the CoA is shown in Figures 6B,C. In general, the entire cluster is predominantly marked by pallial genes, including *Tbr1* and *Emx1* (Gorski et al., 2002; Medina et al., 2004; Tole et al., 2005; García-López et al., 2008; Hirata et al., 2009). Similar to other pallial tissue, scattered interneurons can be found throughout these nuclei (Nery et al., 2002; Real et al., 2009; Vucurovic et al., 2010; Puelles et al., 2016b).

Before the establishment of the radial unit model, the principal cells of the ACo were ascribed to the VP based on *Dbx1*-lineage tracing (non-overlapping with GABA) and its expression of *Tbr1*, *Sema5A*, *Ngn2*, and *Lhx9* (Medina et al., 2004; García-López et al., 2008; Hirata et al., 2009; Bupesh et al., 2011b; Puelles et al., 2016a). ACo *Dbx1*+ cells are generated 1 day before *Dbx1*+ cells of the BLC complex (E10.5 vs. E11.5) and preferentially inhabit the posterior ACo (Hirata et al., 2009; Puelles et al., 2016a). A second population of *Tbr1-*expressing neurons is not derived from the *Dbx1*-lineage, and likely overlaps with *Emx1*-lineage cells (Gorski et al., 2002; Puelles et al., 2016a). The principal cells of the ACo are now understood to be of anterior pallial amygdalar origin, one of the regions within the amygdalar pallial tissue that shares some genetic markers with the ventral pallium (Garcia-Calero et al., 2020). A small subset of hypothalamic-derived TOH cells can also be found in the ACo (Morales et al., 2021). Comparable to the BLC, the ACo nucleus shows aberrant morphology and positioning in *Pax6*^sey/sey^ mice (Tole et al., 2005).

Both subdivisions of the PCo were also found to contain Emx1-lineage cells, and were suggested to be of VP, LP, DP, or MP origin (Puelles et al., 2000; Gorski et al., 2002; Medina et al., 2004; Cocas et al., 2009). Previous findings that the PLCo and PMCo only contained a marginal amount of *Dbx1*-lineage cells were later rectified, and both nuclei were reinterpreted to contain *Dbx1*-progeny (Puelles et al., 2016a; Garcia-Calero et al., 2021). The progenitor region of the PLCo is thus allocated to the basal amygdalar unit, while the progenitor zone of the PMCo is located within the posterior unit, as expected based on the strong expression of *Lhx9* (Garcia-Calero et al., 2020; Garcia-Calero and Puelles, 2021). Some SPV-derived neurons (*Otp*+) were also identified in the PMCo, although it is currently unknown whether they comprise the population originating from the TOH (co-expressing *Foxg1*) or an *Otp*+ cell population from another SPV domain (García-Moreno et al., 2010). Since their presence within the PMCo was not mentioned in any of the more recent papers, it is possible that the ACo and PMCo population is the same, and the nuclei had been wrongly annotated.

*Dlx5/6*-expressing interneurons in the CoA are likely from both CGE and MGE origin, as homotopic transplantation studies found that CGE and MGE grafted cells overlapped in the CoA (Nery et al., 2002; Wang et al., 2011). A CGE “proper” origin was also corroborated by the transplantation of E14.5 CGE-derived *5HT3aR*:GFP+ cells, which colonized the CoA (Vucurovic et al., 2010). The strong expression of *CoupTf2* in the CoA at P0 supports a partial CGE origin (Tang et al., 2012). In addition, scattered *Nkx2.1*-lineage interneurons can be found throughout the CoA, a subset of these cells in the PLCo/PMCo are likely generated in the cvMGE, as evident from their early expression of *Sst* (Xu et al., 2008; Real et al., 2009; Puelles et al., 2016b).

##### Amygdalo-Hippocampal Interface

The AHi was originally often classified within the “transition areas” of the amygdala, however, due to the presence of a distinct boundary between the AHi and hippocampus and its similarity to PMCo, we have opted to discuss this nucleus here instead. Cells within this caudal transition area are marked by the expression of *Lhx9*, *Sema5A*, and *Ngn2* (Medina et al., 2004). Puelles et al. (2016a) and Garcia-Calero and Puelles (2021) recently remarked that an earlier observation that the AHi lacks *Dbx1*+ cells was faulty. *Emx1*-lineage cells can also be found within this area (Gorski et al., 2002). The AHi represents the periventricular part of the posterior radial amygdala unit, its cells therefore likely originate in the ventral ventricular zone at the caudal end of the hemisphere (see Figure 6D). Conditional deletion of *CoupTf2* (Rx-Cre) results in the extensive loss of the AHi (Tang et al., 2012). *Sst*-expressing cells originating from the cvMGE are also present in this nucleus, and appear to be a caudoventral extension of the cvMGE-derived migratory stream toward the BSTia, LA, and BMA (see section Diagonal Area) (Puelles et al., 2016b).

#### Other Amygdalar Nuclei

The “other amygdalar nuclei” comprise amygdalar transition areas and cell clusters that have been the source of controversy regarding their ontogeny.

##### Intercalated Cells

The ITCs are located in paracapsular cell clusters (pcITCs) surrounding the BLC and in the more ventrally located main intercalated cell mass (IA). The ITCs are sometimes considered a part of the central extended amygdala, based on their shared molecular markers and probable developmental origin, as further explained below. However, ITCs are not as homogenous as previously assumed, explaining their classification within the “other” amygdalar nuclei.

A striatal character of the ITCs was suggested based on gene expression analysis (lack of *Tbr1*, weak *Nkx2.1*, *Lhx6* expression and strong *Dlx5* expression) (Medina et al., 2004; García-López et al., 2008; Kaoru et al., 2010). Moreover, the ITCs express *Gsh2* and *Pax6*, indicating that they are derived from the LGE (Tole et al., 2005; Waclaw et al., 2010; Bupesh et al., 2011a; Cocas et al., 2011). Indeed, conditional *Gsh2* LOF (Foxg1tTA/+) mice show reduced numbers of ITCs in both the IA and lateral pcITC (Waclaw et al., 2010; Kuerbitz et al., 2018). The dLGE identity was further proven by means of conditional *Sp8* LOF (Dlx5/6-Cre) and lineage tracing of *Isl1*. As expected, the phenotype observed in the conditional *Gsh2* LOF mouse was mimicked in its *Sp8* counterpart, while no *Isl1*-lineage cells could be observed in the IA or pcITCs (Waclaw et al., 2010). Conditional LOF of *Pax6* within the *Gsh2*-lineage resulted in a strong reduction of the ITCs, highlighting the necessity of *Gsh2* and *Pax6* co-expression for the correct specification of ITCs (Cocas et al., 2011).

Conditional LOF (*Dlx1*-Cre lineage) of *Tshz1*, a gene downstream from *Sp8* that is expressed in migrating dLGE cells, exhibit a complete loss of ITCs in the lateral pcITC and in the IA at E18.5-P3, while cells of the medial pcITC were strongly reduced at P3, but not at E18.5 (Kuerbitz et al., 2018). Postnatal progeny of *Tshz1*KO cells were found to have switched cellular fates and upregulated *Foxp1* (striatal projection neuron marker; Tamura et al., 2004; Precious et al., 2016) instead of *Foxp2* (ITC marker; Kaoru et al., 2010; Waclaw et al., 2010; Kuerbitz et al., 2018). A strong co-labeling of cleaved caspase-3 and GFP was observed in the amygdala of conditional mutant animals (P0.5), indicating that loss of *Tshz1* expression results in increased apoptosis (Kuerbitz et al., 2018). This effect was a direct result of loss of *Foxp2* expression, as *Foxp2*^–/^*^–^* mice recapitulated this phenotype (Kuerbitz et al., 2018).

A small subset of ITCs was found to strongly express *Tbr1* and lacked expression of subpallial markers (Medina et al., 2004). These cells likely overlap with the scarce *Emx1*-lineage cells identified by Gorski et al. (2002). Remedios et al. (2007) hypothesized that a subpopulation of cells generated in the posterior amygdala radial unit (in the original paper called the cvDP) migrate toward the intercalated cell masses, although no other papers have confirmed the presence of nLOT2 cells within the ITCs.

##### Anterior Amygdala Area

The anterior amygdaloid area (AAA) consists of two subdivisions, termed the dorsal and ventral AAA (AAd and AAv, resp.). A schematic overview of its developmental origin is shown in Figure 6A. Both subdivisions were found to be mostly devoid of *Emx1*-expressing cells (Gorski et al., 2002). Gene expression analysis confirmed that the AAd is molecularly similar to the BSTL, and could be assigned a “striatal identity” based on the strong expression of *Dlx5, Pax6, Lmo4*, weak expression of *Nkx2.1*, *Lhx6* and lack of *Tbr1* expression (Stoykova et al., 2000; Medina et al., 2004; Tole et al., 2005; García-López et al., 2008). Cell tracking studies confirmed that the AAd contains a significant proportion of dLGE-derived *Pax6*-expressing cells, which explains its aberrant morphology in *Pax6*^sey/sey^ mice (Stoykova et al., 2000; Tole et al., 2005; Bupesh et al., 2011a).

García-López et al. (2008) postulated that the AAv is primarily derived from the cvMGE based on the expression pattern of *Lhx6* and *Npy*. However, the AAv contains only a very limited amount of *Sst-*expressing cells (García-López et al., 2008; Puelles et al., 2016b). Moreover, the AAv seems developmentally related to the adjacent ACo based on its expression of *Dbx1*, *Emx2, Lhx2*, and *Lhx9* (Tole et al., 2005; Puelles et al., 2016a; Garcia-Calero and Puelles, 2021). Indeed, while the AAA is generally considered a subpallial region, several *Lhx9*+ cells from the anterior pallial amygdala unit were found to invade the AAA (Garcia-Calero et al., 2020). Some *Otp*+/*Foxg1*+ cells from the hypothalamus (TOH) were also identified in the AAv (Morales et al., 2021). Similar to the AAd the AAv is malformed in *Pax6*^sey/sey^ mice (Stoykova et al., 2000; Tole et al., 2005).

### Migration

Migration of neurons from their place of birth toward their final destination is a critical process during embryonic development. This is encoded by cell-intrinsic genetic programs that provide these cells with a repertoire of receptors that can react to the molecular environment of the developing brain. Migrating neurons will scout this environment *via* (leading) processes equipped with these receptors. By continuously changing the orientation of their processes, a preferential migratory route is established, either *via* repulsion or attraction. Different migratory mechanisms have been described, including tangential migration, radial glial-guided migration, and chain migration (reviewed in Marín and Rubenstein, 2001; Nadarajah and Parnavelas, 2002; Valiente and Marín, 2010; Guo and Anton, 2014; Hatanaka et al., 2016; Hirota and Nakajima, 2017). Migration allows progenitors from spatially distinct progenitor pools to intermix, thereby increasing the neuronal heterogeneity and functional potential of brain regions. Here, we will describe findings on previously identified migratory mechanisms and routes from hypothalamic, pallial and subpallial progenitor zones toward the different nuclei of the amygdala.

#### Caudal Ganglionic Eminence

**FIGURE 7:**
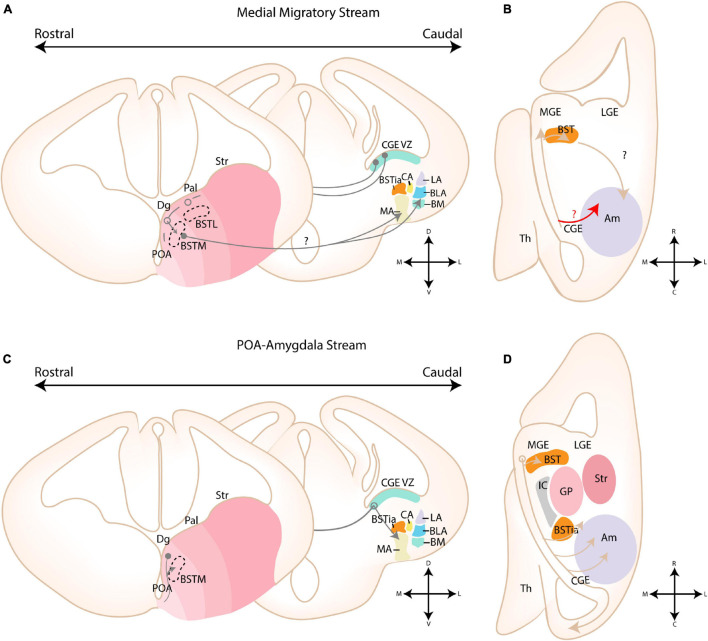
Migratory routes of cells through the medial and POA-amygdala migratory streams. **(A)** Schematic coronal sections showing the putative migratory route of CGE-derived cells within the medial migratory stream. 5HTaR-expressing interneurons originating in the ventricular zone of the CGE (CGE VZ) take a rostral route toward the MGE, where they invade the medial part of the bed nucleus of the stria terminalis (BSTM). Cells from this migratory route are hypothesized to colonize the amygdala, although a migratory route beyond the MGE has not been described. **(B)** Schematic horizontal section showing the putative migratory route of CGE-derived cells within the medial migratory stream. A red arrow shows an alternative migratory route of CGE-derived interneurons destined for the amygdala. **(C)** Schematic coronal sections showing the migratory route of POA-derived cells within the POA-Amygdala migratory stream (PAS). Cells originating in the preoptic area (POA) first migrate dorsally toward the caudoventral MGE/Diagonal area (cvMGE/Dg) region, where a subset of cells tangentially invades the BSTM, while other cells continue their migration caudally. At the level of the CGE, a subset of POA-interneurons migrate ventrally toward the primordium of the developing medial amygdala. **(D)** Schematic horizontal section showing the migratory route of POA-derived cells within the POA-Amygdala migratory stream. A section at the level of the Dg is shown, more dorsal than the POA. POA-derived interneurons first migrate dorsally toward the Dg area, after which they take a caudal route toward the CGE. At the level of the ventral CGE, POA-derived interneurons migrating through the PAS are joined by CGE-derived interneurons migrating through the caudal migratory stream (CMS). While a subset of POA- and CGE-derived interneurons invade the amygdala, others continue along the CMS toward the hippocampus. Str, Striatal domain; Pal, Pallidal domain; Dg, Diagonal Domain; POA, Preoptic Area; BSTL, lateral part of BST; BSTM, Medial part of BST; BSTia, intraamygdaloid part of BST; MA, medial amygdala; CA, Central amygdala; LA, Lateral amygdala; BLA, Basolateral amygdala; BM, Basomedial amygdala; Th, Thalamus; IC, Internal Capsule; GP, Globus Pallidus. Positions where cells go in the slide are shown with a filled circle, positions where cells come out of the slide are shown with an open circle.

**FIGURE 8:**
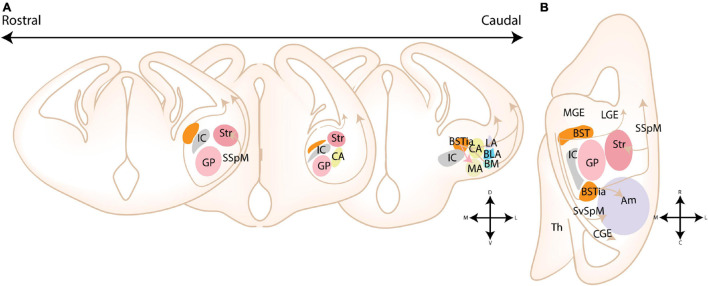
Migratory routes of cells originating in the Diagonal area. **(A)** Rostrocaudal gradient of schematic coronal sections showing the migratory routes of caudoventral MGE/Diagonal area (cvMGE/Dg)-derived cells toward the cortex, striatum and amygdala. At rostral levels, the early-born Dg-derived cells selectively surround the Globus Pallidus (GP), after which cells migrate subventricularly (smaller population, smaller arrow) or superficially toward the striatum and cortex. At more caudal ends of the telencephalon, a presumed radially migrating stream of Sst cells originating in the intraamygdaloid part of the BST (BSTia) passes behind the GP, through the developing CA, thereby connecting to the SSpM laterally. An intraamygdaloid migratory stream tangentially colonizes the pallial amygdala starting from the BSTM(ia), passing the BLA dorsally and caudally, connecting rostrally and dorsolaterally with the LA and caudoventrally with the BMA. A continued migration connects this stream to the AHi (not shown). A migration route from the bed nucleus of the stria terminalis (BST) toward the medial amygdala (MA) is also shown (pink arrow), although this migration route is still controversial. **(B)** Schematic horizontal section showing the migratory routes of caudoventral MGE/Diagonal area (cvMGE/Dg)-derived cells toward the cortex, striatum and amygdala. The subpial subpallial migratory stream (SSpM) and the subventricular subpallial migratory stream (SvSpM) are marked. In addition to the routes mentioned in panel **(A)**, the AHi is also invaded, either from the SvSpM (shown) or medially from the intraamygdaloid stream (not shown). Str, Striatal domain; Pal, Pallidal domain; Dg, Diagonal Domain; POA, Preoptic Area; BSTL, lateral part of BST; BSTM, Medial part of BST; BSTia, intraamygdaloid part of BST; MA, medial amygdala; CA, Central amygdala; LA, Lateral amygdala; BLA, Basolateral amygdala; BM, Basomedial amygdala; Th, Thalamus; IC, Internal Capsule; GP, Globus Pallidus; SSpM, subpial subpallial migratory stream; SvSpM, subventricular subpallial migratory stream.

A unique tangential migratory stream from the CGE toward the amygdala has been recently identified *via* three independent tracing methods (*5HT3aR* reporter mouse line, homotopic transplantation and cell tracker components) (Touzot et al., 2016). This novel migratory stream was termed the “medial migratory stream” (MMS) (see Figures 7A,B). The use of *ex vivo* brain slices in combination with cell tracker components and immunohistochemistry confirmed that these *5HT3aR*-derived cells are likely from CGE origin, and not POA or cvMGE/Dg origin, based on their lack of *Nkx2.1* expression (Touzot et al., 2016). CGE-derived *5HT3aR*-expressing neurons were found to migrate rostrally into the MGE, crossing the BST, and ultimately colonizing the BLC and MA (see Figures 7A,B; Touzot et al., 2016). This stream is most prominent between E13.5-15.5, and by E18.5 the majority of CGE-derived cells have reached the amygdala (Touzot et al., 2016). Unfortunately, neither presence of labeled cells in the CoA and CA, which were previously suggested to contain CGE-derived interneurons, nor the migratory route beyond the MGE, toward the amygdala, was investigated. The authors proposed that the MMS supplies the amygdalar CGE-derived interneurons based on CGE transplantation experiments, the time point during which migration through the MMS and the expression of *CoupTf2* peaks (Touzot et al., 2016). Nonetheless, an initial rostral migration toward the MGE, followed by a caudal migration toward the amygdala seems contradictory, as visible on Figures 8A,B. While we do not doubt the existence of the migration stream from CGE to MGE, which was beautifully illustrated in their paper, it could be possible that this migration route is used by CGE interneurons that have to migrate to more rostral domains, such as the BST. CGE-derived interneurons that are destined for more caudal amygdalar nuclei could instead directly migrate from the CGE toward the amygdala, shown in Figure 8B with a red arrow, a route that is known to be permissible for interneurons (compare with Figure 7D for POA-derived interneurons and Figure 8B for Dg-derived interneurons). Possible experiments that could further investigate this issue include *ex vivo* life slices, lightsheet imaging to better visualize the migrating *5HT3aR*-interneurons in 3D and transplantation studies of the MGE region containing CGE-derived *5HT3aR*-interneurons.

Migrating cells within the MMS are characterized by their expression of *CoupTf2*, *Sp8*, and *Prox1*, although at a lower level than reported in other CGE-derived migratory streams (Yozu et al., 2005; Kanatani et al., 2008; Touzot et al., 2016). In conditional *CoupTf1* LOF mutants (*Dlx5/6*-Cre lineage), the number of *CoupTf2*-expressing cells migrating in the MMS is decreased at E13.5, but significantly increased at E15.5. *CoupTf1* plays a dual role in the CGE, as it regulates the proliferation of intermediate progenitor cells in the CGE and the number of migratory cells within the MMS, the latter *via* control of *CoupTf2* expression (Faedo et al., 2008; Lodato et al., 2011; Touzot et al., 2016). The increase of proliferating cells in SVZ is reflected by an initial decrease of cells migrating early, at E13.5. Subsequently, at E15.5, an increased amount of post-mitotic cells start their migration, explaining the higher numbers observed at this embryonic stage (Touzot et al., 2016). *CoupTf2* was previously found to regulate the expression of Semaphorin receptors *Nrp1/Nrp2* in the embryonic brain, two molecules that are known to modulate migration and axon guidance during development (Marín et al., 2001; Tamamaki et al., 2003; Cariboni et al., 2007; Chen et al., 2007; Le et al., 2007; Nóbrega-Pereira et al., 2008; Tang et al., 2012; Kanatani et al., 2015). Expression of *CoupTf2* was found to increase in absence of *CoupTf1*, conditional loss of *CoupTf1* expression might therefore indirectly affect the migration of these cells by increasing *Nrp1* and *Nrp2* expression *via CoupTf2* (Tang et al., 2012; Flore et al., 2016; Touzot et al., 2016). In addition, *CoupTf2* has been shown to guide migrating (inter)neurons within the CMS and other routes toward the amygdala (Yozu et al., 2005; Kanatani et al., 2008, 2015; Tang et al., 2012; Cai et al., 2013). In the neocortex, *5HT3aR* expression is responsible for the proper positioning of CGE-derived interneurons (Murthy et al., 2014). Whether similar mechanisms govern the positioning of CGE-derived cells within the amygdaloid complex is currently unknown.

#### Preoptic Area

Via *Dbx1*-lineage tracing, Hirata et al. (2009) identified a migratory stream that they termed the “POA-amygdala migratory Stream (PAS)” (see Figures 7C,D). These POA-derived cells were speculated to migrate along radial glial fibers, based on their close proximity to RC2-positive fibers at E13.5 (Hirata et al., 2009). Whole mount electroporation of this region by Kanatani et al. (2015) confirmed the existence of this migratory stream. *In utero* electroporation experiments of E11.5 embryos revealed that these cells migrate in a narrow stream through the cvMGE and vCGE, after which they spread out to inhabit the MA and the cortex. During their migration through the vCGE, POA-derived cells intermingle with migrating CGE cells from the caudal migratory stream (CMS) (Kanatani et al., 2015). Migration through the PAS was found to be highly dependent on *CoupTf2* expression, as overexpression of *CoupTf2* at E11.5 resulted in a preferential colonization of the MA vs. the cortex at E15.5. Moreover, the cells migrated along a more narrow path through the vCGE. In contrast, knockdown of *CoupTf2* resulted in scattering of CMS/PAS cells throughout the ventral telencephalon, severely reducing the population in the MA and instead increasing the population that reached the cortex. *CoupTf2* expression is therefore necessary for all cells in the early PAS/CMS, but is downregulated by cells migrating to the cortex and maintained by cells migrating to the MA (Kanatani et al., 2015). *Nrp2* was identified as a downstream target of *CoupTf2* expression in the PAS, and effects of *CoupTf2* knockdown could be rescued by *Nrp2* overexpression (Kanatani et al., 2015). The striatum is known to strongly express *Sema3A* and *Sema3F* at embryonic ages, and the repulsive interaction between these molecules and their *Nrp1/Nrp2* receptors is known to guide MGE-derived cortical interneurons around the striatum toward the cortex (Marín et al., 2001; Tamamaki et al., 2003; Cariboni et al., 2007; Chen et al., 2007; Le et al., 2007; Nóbrega-Pereira et al., 2008). Perhaps POA interneurons utilize similar mechanisms, where the preservation of *CoupTf2/Nrp2* expression results in complete avoidance of the striatal area, leading them toward the MA. While Kanatani et al. (2015) did not focus on the BST, Bupesh et al. (2011b) described a tangential migration from the POA toward the BSTM, as shown in Figures 7C,D.

#### Diagonal Area

Several reports have emerged about the route of migrating cvMGE/Dg-derived cells toward the cortex and amygdala (see Figure 8). Cell tracking (CMFDA) of cvMGE/Dg-derived cells on *ex vivo* live slices at E13.5-E16.5 by Bupesh et al. (2011b) identified migration of cvMGE cells toward the BSTM, MeA and MePD. Based on the parallel orientation of their leading processes to the radial glial fibers, they concluded that cvMGE-derived cells might migrate along glial fibers toward the BSTM (Bupesh et al., 2011b). Moreover, the BSTL, CeL, and ACo were also labeled, but in contrast the leading processes of migrating cells were oriented orthogonal to the radial glial fibers, indicating tangential migration (Bupesh et al., 2011a,b). The PMCo/PLCo, AAA, and AHi were not mentioned in this paper. Later findings from this research group indicated that the BSTM lies within the radial domain of the TOH, and thus migration from cvMGE toward this nucleus is likely tangential (Morales et al., 2021). Since multiple migratory routes seem to pass through the cvMGE/Dg, the results of Bupesh et al. (2011b) should be carefully interpreted. While the majority of labeled cells within the CeL expressed *Sst* from an early stage, co-expression analysis was not performed for the other nuclei. Whether they truly originated in the cvMGE should therefore be further investigated, especially since POA-derived interneurons that invade the MA pass through the cvMGE on their way to the amygdala (Kanatani et al., 2015).

Multiple migratory paths originating in the Dg were identified, including a superficial subpallial and a subventricular subpallial migratory route (termed SSpM and SvSpM, resp.), similar to those taken by MGE-derived cortical interneurons (Puelles et al., 2016b). While the subpial migratory stream is apparent from E10.5 onward, the first *Sst*-expressing cells in the subventricular stream only appeared at E12.5 (Puelles et al., 2016b). Analysis of (early-born) *Sst*-expressing neurons by García-López et al. (2008); Real et al. (2009) and Puelles et al. (2016b) showed a gradual spread from the ventricular zone of the cvMGE/Dg, selectively surrounding the Globus Pallidus after which cells take a tangent toward the striatum and cortex. At more caudal ends of the telencephalon, a presumed radially migrating stream of *Sst*+ cells originating in the BSTia passes behind the GP (through the primordium of the developing CA), thereby connecting to the SSpM laterally (García-López et al., 2008; Puelles et al., 2016b). An intra-amygdaloid migratory stream tangentially colonizes the pallial amygdala starting from the BSTM(ia), passing the BLA dorsally and caudally, connecting rostrally and dorsolaterally with the LA and caudoventrally with the BMA (Puelles et al., 2016b). A continued medial migration is hypothesized to connect this stream to the AHi, although the AHi could alternatively be invaded from the SvSpM (Figure 8B; Puelles et al., 2016b). The migratory population toward the BSTMa and CeL was described as radial, in contrast to findings by Bupesh et al. (2011b), while the migratory population to the pallial amygdala is tangential (Bupesh et al., 2011b; Puelles et al., 2016b). Puelles and colleagues mention that their analysis at an earlier stage was likely instrumental for the detection of this radial population. While the molecular cues that guide these cells to the amygdala have not been investigated, the authors remarked that Dg-derived cells completely avoided the POA area and the pallidum, indicating that repulsive mechanisms might restrict these cells to the Dg histogenetic division (Puelles et al., 2016b).

#### Lateral Ganglionic Eminence

##### Ventral Lateral Ganglionic Eminence

**FIGURE 9:**
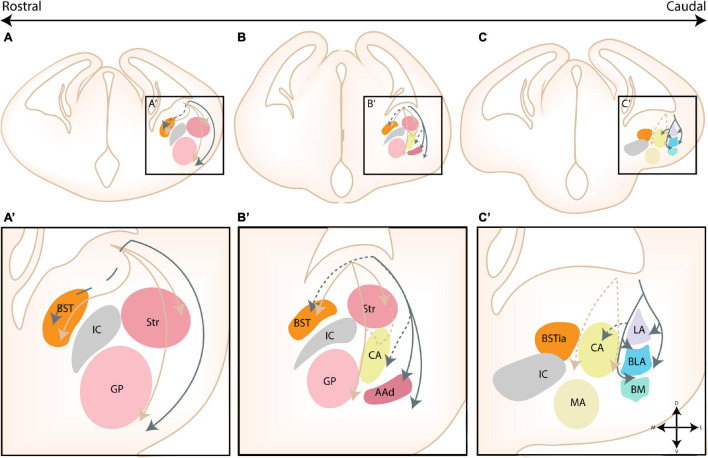
Migratory routes of cells originating in the dorsal and ventral LGE. **(A–C)** Rostrocaudal gradient of schematic coronal sections showing the migratory routes of dorsal and ventral (dLGE/vLGE)-derived cells toward the striatum (Str) and amygdala. **(A’–C’)** zoom in of squares in panels **(A–C)**. **(A,A’)** At rostral levels, cells originating from the vLGE (brown arrows) migrate radially toward the striatum or tangentially, either toward the bed nucleus of the stria terminalis (BST) or *via* a route that passes the Str ventromedially. dLGE-derived neurons (gray arrows) instead migrate toward the olfactory cortices. A hypothesized tangential migration (striped line) delivers dLGE-derived cells to the BSTL. **(B,B’)** At intermediate levels, the three migratory routes of vLGE-derived interneurons can be visualized. The central amygdala (CA) receives cells from the vLGE, although it remains unclear whether this migratory stream is an extension of the radial migratory stream toward the Str or the stream that passes the Str ventromedially (possible migratory routes toward CA shown with striped lines). The hypothesized route of dLGE-derived cells toward the olfactory cortices and BST, respectively, can be visualized at intermediate levels. A subset of cells from the VMS also invade the AAd. The CA is also hypothesized to be invaded by dLGE-derived cells (striped line). **(C,C’)** At more caudal levels, two putative migratory streams from the vLGE, surrounding the CA, are shown with striped lines. These are hypothesized caudal extensions of the radial and tangential migratory routes of vLGE-derived neurons that might deliver cells toward the CA at more rostral levels. dLGE-derived cells migrate around the developing basolateral complex (BLC), thereby contributing to the intercalated cell masses that surround this complex. This medial stream around the BLC stream might additionally deliver cells to the CA (hypothesized, striped lines). AAd, dorsal part of anterior amygdala; Str, Striatal domain; BST, bed nucleus of the stria terminalis; BSTia, intraamygdaloid part of BST; MA, medial amygdala; CA, Central amygdala; LA, Lateral amygdala; BLA, Basolateral amygdala; BM, Basomedial amygdala; IC, Internal Capsule; GP, Globus Pallidus.

*Ex vivo* migration assays with cell tracker components (CMFDA) and gene expression analysis (*Isl1*) at E13.5–E16.5 by Bupesh et al. (2011a) revealed that vLGE-derived cells tangentially invaded the BSTL medial to the internal capsule. This finding was corroborated by Tinterri et al. (2018), who investigated the tangential migration of corridor cells from the vLGE in detail (see Figure 9). Corridor cells tangentially migrated into the MGE territory around E13.5, after which a subset contributed to the BST (Tinterri et al., 2018). Conditional LOF of *Ebf1* (*Dlx5/6*-Cre lineage), which show abnormal migration of corridor cells, reduced the amount of “corridor cells” within the BST and affected the total size of the BST at postnatal ages (P5) (Tinterri et al., 2018). Another tangential migratory stream that passes the striatum ventromedially and delivers cells to the interstitial nucleus of the posterior limb of the anterior commissure (Ipac), was identified (Tinterri et al., 2018). The anatomical location of the Ipac vs. the CA could imply that this migratory route also delivers cells to the CA. Data from Bupesh et al. (2011a) suggests that the vLGE-derived cells of the CA are either a ventrolateral extension of the radial vLGE-derived migratory stream toward the striatum, or tangential derivatives of the ventromedial migratory route described by Tinterri et al. (2018). However, more research is required to fully understand the migratory route of these cells.

##### Dorsal Lateral Ganglionic Eminence

The PSB separates the progenitor domains of the subpallial dLGE and the pallial VP primordia; the VP gives rise to the Ventropallial Migratory Stream (VMS), often referred to as the Lateral Cortical Stream (LCS) in earlier publications. The migration of these populations are temporally controlled, as the onset of migration of VMS *Pax6*+/*Dlx2-* cells (E11.5) occurs before dLGE *Dlx2*+ cells (E13.5) (Carney et al., 2006; Hirata et al., 2009).

In contrast to the pallial VMS, only few *Dlx2-*expressing, dLGE-derived cells appeared to contact the radial glia (Carney et al., 2006). Instead, the majority of the subpallial dLGE-derived population seems to migrate *via* chain-migration and exits the cell cycle only after the onset of their migration, similar to dLGE-derived cells of the rostral migratory stream (RMS) (Carney et al., 2006). Loss of *Pax6* expression was found to severely affect the amount of migrating dLGE-derived cells, likely as a consequence of a migratory arrest (Tole et al., 2005). Based on data from Carney et al. (2006); Bupesh et al. (2011a), and Kuerbitz et al. (2018), the *Dlx2*+ and *Tshz1*+ cells derived from the dLGE seem to migrate around the developing BLC, thereby contributing to the intercalated cell masses that surround this complex (see Figure 9). Some cells also seem to invade the BLC. A significant part of the migration stream then continues their migration toward the AAd (see Figure 9; Bupesh et al., 2011a). How dLGE cells reach the CA and BSTL is currently not well-understood, but they are hypothesized to be tangential migrations from a stream splitting from the dLGE (BSTL), or the stream surrounding the BLC (CA) (Bupesh et al., 2011a).

Migration of dLGE-derived cells destined for the ITCs was found to be (partly) dependent on *Tshz1* expression (Kuerbitz et al., 2018). While the main function of *Tshz1* is regulation of ITC differentiation and survival, several genes involved in guidance and migration showed abnormal expression levels in the conditional LOF (*Dlx1*-Cre lineage), including *ErbB4*, *Prokr2*, and *Dcc* (Kuerbitz et al., 2018). Downregulation of the Neuregulin receptor *ErbB4* was confirmed with immunostaining and is linked to the regulation of MGE-derived cortical interneuron and dLGE-derived olfactory bulb interneuron migration (Yau et al., 2003; Anton et al., 2004; Flames et al., 2004; Kuerbitz et al., 2018). Downregulation of *ErbB4* might affect the migration of cells through the permissive *Nrg1* corridor in the LGE, and therefore confine these cells to the migratory stream (Flames et al., 2004). Within the RMS, *Tshz1* was previously shown to be critical for the transition from chain to radial migration (Ragancokova et al., 2014). In contrast to the ITCs, the CA seems devoid of *Tshz1*-derived cells (Kuerbitz et al., 2018).

#### Pallial Amygdala

##### Lessons From the Ventropallial Migratory Stream

The pallial amygdalar cells were originally believed to reach their amygdala destinations *via* the ventropallial migratory stream (VMS). It was recently discovered that the amygdalar pallial field is positionally separated from the cortical pallial field (Puelles et al., 2019; Garcia-Calero et al., 2020; Garcia-Calero and Puelles, 2021), complicating these observations. Moreover, while cortical pallial cells mainly utilize radial glia-guided migration, the amygdalar pallial nuclei are generated *via* outside-in cell aggregation (Molnár and Butler, 2002; Carney et al., 2006; Bai et al., 2008; Garcia-Calero et al., 2020). Below, we provide an overview of some molecules that might be relevant for the amygdala.

Similar to structures derived from the VP, no gross developmental abnormalities in amygdala structures have been described in Reeler or *Cdk5*^–/^*^–^* mice, with the exception of the nLOT2 (see below) and a single cell population in the MePD, of which the origin has not been identified (Remedios et al., 2007; Boyle et al., 2011). The apparent migration of VP-derived cells of the VMS (*Tbr1*+) was found to be dependent on the expression of *Dcx* and *Lis1*, two genes that are known to be involved in migration (Bai et al., 2008). Transfer of *Dcx* shRNA to the VP at E13 caused cells to be stuck within the migratory stream, apparently unable to invade the surrounding amygdalar tissue (Bai et al., 2008). As we now understand that VP cells do not invade the amygdala and are restricted to the olfactory cortices (Garcia-Calero and Puelles, 2021), we can deduct that their *in utero* electroporation also included pallial amygdalar progenitors, which are therefore also dependent on *Dcx* expression for correct migration. Alternatively, non-amygdalar tissue might have been misinterpreted as amygdalar. Likewise, knockdown of *Lis1* decreased the amount of migrating cells, and cells that did migrate got arrested in white matter tissue (Bai et al., 2008). The morphology of the cells was also severely affected; *Dcx* RNAi resulted in shorter leading processes, while those of cells treated with *Lis1* RNAi were significantly longer (Bai et al., 2008). In addition, *Lis1*- and *Dcx*-RNAi increased the occurrence of multipolar cells by two and fourfold, respectively (Bai et al., 2008). *Lis1* might also play a role in neuronal positioning and normal synaptogenesis in the amygdala, as evident from heterozygous *Lis1* mutant mice (Toba et al., 2013).

##### The Anterior Pallial Amygdalar Unit

**FIGURE 10:**
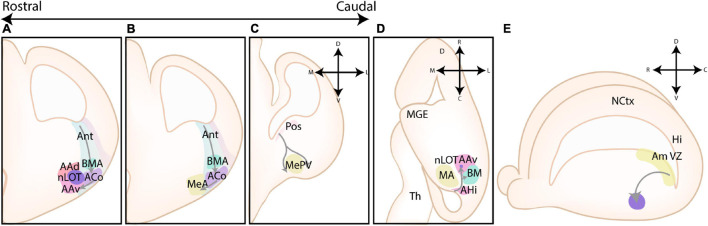
Overview of several migratory routes toward pallial amygdalar nuclei. **(A,B)** Rostrocaudal gradient of schematic radial sections showing the radial translocation of anterior pallial amygdalar unit cells toward its derivatives. Cells from the anterior pallial amygdalar unit, marked by expression of Lhx9, experience massive translocation that completely depletes the periventricular stratum of cells. Migrating cells colonize the anterior basomedial amygdala (BMA) and the anterior cortical amygdala (ACo). From here, cells tangentially invade the anterior amygdala (AA) and the anterior and posteroventral part of the medial amygdala (MeA, MePV, resp.). **(C)** Schematic radial section highlighting the position of the posterior amygdalar unit and the caudoventral pallial (CVP)-derived migratory stream. The origin of this migratory stream seems to be located in the posterior amygdalar unit. This migratory stream splits into two streams that course ventrally to surround the MePV. **(D)** Schematic horizontal section showing the first phase (rostralwards) of the nLOT2 migratory stream (nLOT2ms). Cells originating in the rostromedial part of the amygdalo-hippocampal interface (AHi) course rostrally past the BMA toward the AA. A small stream of cells diverges from the nLOT2ms and seems to invade a medial shell surrounding the BMA. Within the AA, the migration continues ventralwards toward the nLOT2 (not shown). This migratory stream includes posterior pallial amygdalar cells and hypothalamic-derived cells, the migration route of the latter toward the AHi is shown in Figures 11A,B. **(E)** A side view of the developing murine brain, showing the lateral ventricle, the amygdalar ventricular zone (Am Vz) at the level of the posterior amygdalar pallium and the nLOT2 migratory stream (nLOT2ms). Cells originating from this amygdalar radial unit first migrate rostrally, independent from glial fibers, followed by a radial ventral migration toward the nLOT2 (purple). Nctx, Neocortex; Hi, hippocampus; AAd, dorsal part of anterior amygdala; AAv, ventral part of anterior amygdala; ACo, anterior cortical amygdala; AHi, amygdalo-hippocampal interface; BM, Basomedial amygdala MA, medial amygdala; MeA, anterior part of medial amygdala; MePV, posteroventral part of medial amygdala; nLOT2, layer two of nucleus of lateral olfactory tract; Pos, posterior amygdalar pallial unit; Th, Thalamus.

Based on the expression of *Lhx9*, Garcia-Calero and Puelles (2021) reconstructed the gradual development of anterior pallial amygdalar derivatives into intermediate and superficial nuclei (see Figures 10A,B). At early stages of development (E12.5), *Lhx9* labeled the entire radial unit, however, from E14.5 onward, *Lhx9* expression completely disappeared at the periventricular stratum, while the intermediate (BMA) and superficial (ACo) nuclei grew (Garcia-Calero and Puelles, 2021). This finding highlights the unique development of the amygdalar pallial field, which develops in an outside-in instead of inside-out manner, unlike other mammalian pallial cortical regions (Garcia-Calero and Puelles, 2021). A superficial translocation completely depletes the periventricular stratum of cells, which agglomerate at the superficial and intermediate parts of the radial unit (Garcia-Calero and Puelles, 2021). A glial palisade separates the lateral/basal unit from the subpallium at the periventricular level. This massive translocation seems to only occur at the anterior radial unit, as it is the only amygdalar unit that lacks a periventricular part (Garcia-Calero et al., 2020). In addition to its direct pallial derivatives, *Lhx9* positive cells were also found at the level of the AAA, MeA, and MePV, currently postulated to be the result of tangential cell migrations from the ACo (Garcia-Calero and Puelles, 2021). Bupesh et al. (2011b) had previously proposed a tangential migration of “VP-derived cells” toward the MeA and MePV, based on the orientation of the migrating cells and radial glial fibers.

##### Lateral Olfactory Tract Migratory Stream

Cellular migration to nLOT2 was initially characterized by Remedios et al. (2007) using *in utero* electroporation, gene expression analysis and LOF models. This migratory route (shown in Figures 10D,E) originates in the now identified posterior amygdala radial unit and was termed the “Caudal Amygdaloid Stream (CAS)” (Remedios et al., 2007). It was later renamed nLOT2 migratory stream (nLOT2ms) to avoid further confusion about the birthplace of the migrating neurons (Garcia-Calero et al., 2021).

Analysis of *NeuroD* expression, a marker that exclusively labels the nLOT2 and nLOT2ms, predicts the onset of migration around E14.5, and by E16.5 most neurons seem to have arrived in the nLOT2 (Remedios et al., 2007). At the rostromedial AHi, the locally generated nLOT2 cells are joined by hypothalamic *Sim1*+ cells at around E13.5 (see “Hypothalamus”) (Garcia-Calero et al., 2021). These cells then collectively migrate rostroventrally across the pallial amygdala (Garcia-Calero et al., 2021). At E15.5, when the stream is located at the medial side of the BLA and BMA, it crosses into the AAA and continues its migration toward the nLOT2 (Garcia-Calero et al., 2021). A small stream of cells diverge from the nLOT2 and seem to invade a medial shell surrounding the BMA (Garcia-Calero et al., 2021). The nLOT2ms can be subdivided into two phases, including a tangential phase during the initial rostral extension, followed by a radial phase with ventral extension toward the pial surface (Remedios et al., 2007). Garcia-Calero et al. (2021) later deducted that the migratory phases overlap with a switch from migration within pallial tissue to subpallial tissue, respectively.

Intriguingly, cellular migration through the nLOT2ms was found to be dependent on Reelin and *Cdk5* signaling (Remedios et al., 2007). Cells migrating through the nLOT2ms express *Dab1* and form a *Reelin*-negative corridor (Remedios et al., 2007). In Reeler mutant embryos a migratory stream was still visible, however, the route was severely malformed and a “nLOT2-like” structure with aberrant morphology emerged in a more medial and dorsal position along the route (Remedios et al., 2007). In addition, a migratory arrest was found to occur in *Cdk5*^–/^*^–^* mice after the first phase of migration (Remedios et al., 2007). *Zic2* is also expressed in migrating nLOT2 cells, and homozygous deletion of *Zic2* resulted in a less populated and more dispersed migratory stream (Murillo et al., 2015).

##### Caudoventral Pallium and CoupTf2 Migratory Stream

Surprisingly (controversially), an older study by Tang et al. (2012) had identified a CGE-derived migratory stream that supplies glutamatergic neurons toward the BM. As mentioned before, we believe this population constitutes pallial derivatives instead of CGE-derived interneurons and have opted to discuss this migratory route here instead of in section “Caudal Ganglionic Eminence.”

At E13.5, two *Pax6*+ migratory streams were found to arise from the “CGE,” with cells within the medial stream migrating toward the BM (Tang et al., 2012). Similar to cells of the MMS, *CoupTf2* seems to regulate the migration of this cell population, as the number of migratory *Pax6* cells was severely diminished in conditional *CoupTf2* LOF mice (Rx-Cre), without affecting apoptosis or proliferation in the “CGE” (Tang et al., 2012). Immunohistochemical analysis and qPCR assays revealed that the expression of *Nrp1* and *Nrp2* was significantly reduced in the conditional mutant (Tang et al., 2012). *CoupTf*2 was found to directly affect expression of *Nrp1 via* binding of a Sp1 site located within the 12th intron. Similarly, cell tracking experiments (CMFDA) in *ex vivo* slices identified a *Tbr1*+ migratory stream originating in the CVP, seemingly overlapping with the posterior amygdalar unit of Garcia-Calero et al. (2020), that splits into two streams surrounding the MePV (see Figure 10C; Ruiz-Reig et al., 2018). Whether *CoupTf2*-expression also regulates the migration of CVP-derived neurons should be further investigated.

#### Hypothalamus

**FIGURE 11:**
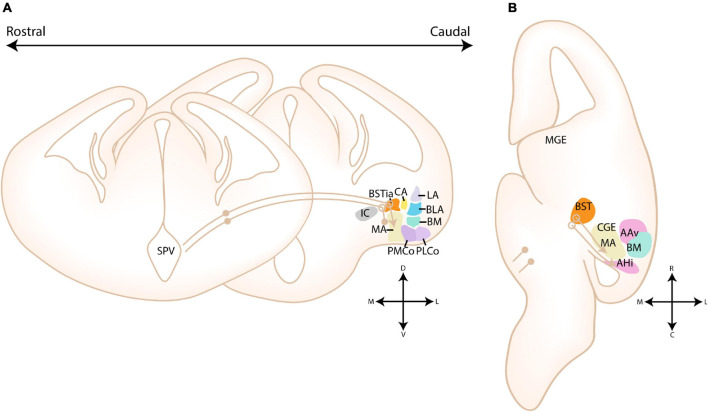
Migration routes from hypothalamic domains toward the amygdala. **(A)** Rostrocaudal gradient of schematic coronal sections showing the migratory routes of supraopto-paraventricular hypothalamus-derived cells toward the amygdala. Two parallel migratory streams, including a rostrodorsal stream originating in the telencephalon-opto-hypothalamic domain (TOH, Otp+/Foxg1+) and a caudoventral stream from more central domains of the supraopto-paraventricular hypothalamus (SPV, Sim1+), emerge from the SPV and course dorsolaterally toward the medial part of the bed nucleus of the stria terminalis (BSTM). After entering the telencephalon, a continued caudoventral migration delivers the TOH-derived cells to the medial amygdala (MA, shown), anterior and/or posteromedial cortical amygdala (ACo, PMCo, resp.) and ventral anterior amygdala area (AAv). The latter two are not visible on the scheme due to their more rostral position. Hypothalamic Sim1+ cells from other SPV domains instead migrate around the MA toward the amygdalo-hippocampal interface (AHi), basomedial amygdala (BMA) and layer two of the nucleus of the lateral olfactory tract (nLOT2). Since this migratory stream courses more caudally, the migration toward AHi, BMA, and nLOT2 is not visible on this scheme. Positions where cells go in the slide are shown with a filled circle, positions where cells come out of the slide are shown with an open circle. **(B)** A schematic horizontal scheme showing the migration from the SPV toward the MA and AHi. Part of the migration route is located more dorsally than the scheme and thus not visible. Sim1+ cells pass the MA and instead migrate toward the AHi, after which they continue their migration toward BMA and nLOT2 as shown in Figures 10D,E. TOH-derived cells instead invade the MA, ACo/PMCo and AAv. Positions where cells go in the slide are again shown with a filled circle, positions where cells come out of the slide are shown with an open circle. AAv, ventral part of anterior amygdala; AHi, amygdalo-hippocampal interface; BLA, basolateral amygdala BM, basomedial amygdala; BST, bed nucleus of stria terminalis; BSTia, intraamygdaloid part of BST; CA, central amygdala; IC, internal capsule; LA, lateral amygdala; MA, medial amygdala; nLOT2, layer two of nucleus of lateral olfactory tract; PLCo, posterolateral cortical amygdala; PMCo, posteromedial cortical amygdala Pos, posterior amygdalar pallial unit; Th, Thalamus.

CFDA injections into the SPV at E10/11 and analysis at E15/18 by García-Moreno et al. (2010) revealed a two-step tangential migratory route of hypothalamic-derived cells toward the MA (García-Moreno et al., 2010). The migratory route is schematically shown in Figure 11. An initial migration delivered the cells to more dorsal hypothalamic regions, followed by migration to enter the telencephalon and a subsequent spread to more caudal regions. Cells first settled within the BSTM, followed by colonization of the MA and PMCo (García-Moreno et al., 2010). These findings were later corroborated with *ex vivo* cell migration assays performed by Bupesh et al. (2011b) and lineage tracing of *Dbx1*-expressing cells from the SPV performed by Puelles et al. (2016a). Morales and colleagues specified that these cells originate in the TOH part of the SVP based on their expression of *Foxg1* (Morales et al., 2021). While a subset of these cells might indeed migrate tangentially, the identification of a radial histogenetic division that extends from the ventricular zone of the TOH toward the caudoventral pial surface of the telencephalon (including the MA) highlights radial migration as a more likely migratory mechanism (Morales et al., 2021).

Garcia-Calero et al. (2021) later termed the collective migration of multiple hypothalamic-derived cells to the amygdala the Hypothalamo-Amygdalar-Corridor (HyA) (Garcia-Calero et al., 2021). This migration stream abuts the stream of Dg-derived cells toward amygdala in the telencephalon and includes two distinct populations, that form a continuum of the paraventricular subdomains previously identified (Garcia-Calero et al., 2021). Indeed, the most dorsal part of the SPV (TOH), mainly delivers cells to subpallial amygdalar areas, while the more central part, which lacks *Foxg1* expression and shows high *Sim1* expression, delivers cells to the nLOT2 and BMA (Garcia-Calero et al., 2021; Morales et al., 2021). Accordingly, the dorsal subdomain extends into the rostrodorsal part of the HyA, while the central part extends into the caudoventral part of the HyA (Garcia-Calero et al., 2021). While the TOH-derived migratory stream seems strongly dependent on *Otp*-expression, the nLOT2 seems unaffected (Wang and Lufkin, 2000; García-Moreno et al., 2010). *Sim1*+ cells from the central SPV enter the telencephalon at the caudal posterior amygdala subunit and migrate laterally, behind the MA, toward the rostromedial AHi where they are joined by the locally generated nLOT2 cells, after which they both migrate toward the nLOT2 and BMA (Garcia-Calero et al., 2021). While the inclusion of TOH-derived cells within the extended medial amygdala might seem logical given its topological position and radial domain, the hypothalamic cells of the BMA and nLOT2 have to first migrate caudomedially around the MA before migrating inside the AHi/PMCo. Recently, a unique *Lhx9*-positive stream of cells, seemingly emerging from the PTE, that passes superficially over the MA and that might reach BAOT has been described, but further confirmation of the place of birth and its destination is necessary (Garcia-Calero and Puelles, 2021).

Knockdown of *Otp* at E10 strongly reduced the amount of TOH-derived cells that reached their destination in the telencephalon, and additionally affected their normal distribution in the hypothalamus (García-Moreno et al., 2010). This reduction was primarily attributed to migratory defects, as knockdown of *Otp* did not influence apoptosis or proliferation (García-Moreno et al., 2010). These migratory defects were mimicked in *Otp*^–/^*^–^* mice, however the molecular mechanism was not investigated (García-Moreno et al., 2010). While migration from the TOH was shown to be dependent on *Otp*-expression, less is known about the migration of SPV-derived cells that lack *Otp*. Some *Sim1*+/*Otp-* cells within the BMA (and CA) seem likewise dependent on *Otp* expression and were absent in the *Otp* mutant, while others were not affected (García-Moreno et al., 2010). This highlights an interesting concept of a non-autonomous function of *Otp*, where cells with different gene expression profiles that migrate through the same corridor are likewise affected. The *Otp*- and *Sim1*-expressing cells have been hypothesized to preferentially target subpallial (*Otp*+) and pallial (*Sim1*+) amygdalar nuclei (García-Moreno et al., 2010; Garcia-Calero et al., 2021; Morales et al., 2021). While this observation seems true for most nuclei, the ACo, a pallial amygdalar nucleus, also receives cells from the *Otp+/Foxg1*+ population (Morales et al., 2021). Moreover, the currently controversial presence of *Sim1*-expressing cells in the subpallial CA contradict this hypothesis (García-Moreno et al., 2010; Garcia-Calero et al., 2021; Morales et al., 2021). A thorough investigation into the function of *Otp* and *Sim1* in these cells, and potential links with their preferential localization, should be carried out. Moreover, it would be interesting to further investigate the function and axonal targets of these hypothalamic cells.

## Discussion and Conclusion

Taken together, it is clear that over the past decennia an enormous effort has been made to elucidate the cellular origins of the amygdalar nuclei. Nevertheless, their complexity has caused a continuous rediscovery of amygdala borders and classification, leaving many questions regarding their exact origin unanswered to this day. Older papers often focused on single gene expression patterns to link nuclei to molecularly “similar” progenitor regions, an at the time common approach that unfortunately resulted in multiple erroneous conclusions. This is highlighted by the only recent discovery that the amygdala is not generated in the cortical pallium, but rather contains its own pallial field. Indeed, the shared presence of some marker genes between cortical pallial and amygdalar pallial regions misled researchers for over two decennia. Examples include the assumption that certain parts of the amygdala must originate from the lateral pallium based on their lack of *Dbx1*-expression and expression of *Emx1* (Puelles et al., 2000; Gorski et al., 2002; Medina et al., 2004; Tole et al., 2005). Moreover, several uncertainties plagued *Lhx9* as a “ventral pallium marker” before its novel role as a specific marker for the anterior and posterior amygdalar radial units (and PTE) was elucidated, such as its presence in some nuclei that were assigned to the VP (e.g., ACo, BM) but its complete absence in other nuclei that were at that time understood to contain cells from VP origin (e.g., LA, BLA) (Remedios, 2004; Tole et al., 2005; García-López et al., 2008). At last, cells of the nLOT2 were reported to originate in a novel caudoventral part of the dorsal pallium, but this progenitor domain is now understood to lie within the posterior amygdalar radial unit (Remedios et al., 2007; Garcia-Calero et al., 2021). With the help of the novel radial histogenetic model of the pallial amygdala, many previously unrelated nuclei share an obvious developmental origin. Indeed, parts of the subpallial and pallial amygdala are strongly interconnected, both through functional connections and developmental plan. For example, pallial cells from the anterior radial unit invade certain subpallial nuclei. *Dbx1*- and *Emx1*-cells were previously assigned to different cortical pallial sectors, but the whole pallial amygdala is now understood to contain a mix of both. Moreover, both conventional “pallial” and “subpallial” nuclei receive hypothalamic-derived cells, although their gene expression profiles might result in a preferential localization of specific cell-types. More recent papers often combined gene expression studies with lineage tracing, gene transfer and transplantation studies. However, some migratory streams pass through other progenitor regions before ultimately merging, complicating conclusions that can be made from these studies.

Another standard practice in developmental studies, not limited to the amygdalar field, includes the use of coronal sections to investigate causal relationships between progenitor zones and their derivatives. This practice was strongly influenced by studies on cortical development, where the ventricular zone and the cortical plate are conveniently located within the same coronal plane. In contrast, during mammalian evolution the amygdala has significantly rotated. In mice the true progenitor zones are therefore located in more caudal sections compared to their derivatives (Garcia-Calero et al., 2020). Consequently, many of the older papers that were based on coronal sections should be reinvestigated. One finding that could be further clarified, either in radial sections or with lightsheet microscopy, is the subtle difference between the LA and BLA, as mentioned in section “Basolateral Complex.” The developmental origin of several other amygdalar nuclei also remain poorly understood and could be further studied, including the nLOT1 and nLOT3, the BAOT, the AAA, the pallial part of the ITCs and the AHi.

The existence of a *CoupTf2-*positive and *Dbx1-*negative caudal pallial amygdalar subdivision is an interesting concept that should be investigated further. Taking into account the radial orientation of the amygdala and its separate pallial field, the expression pattern of *Gdf10* (a CVP marker) seems widespread in more caudal pallial amygdalar ventricular zones. Ruiz-Reig et al. (2018) comment on the unique shape of the VP/CVP in their paper, highlighting the bending of the VP, whereby it seems to surround the CGE on both the lateral ánd medial side. The more “medial” pallial tissue at the level of the CGE expressing *Gdf10*, which forms a continuum with the more lateral pallial tissue at caudal levels, could very well represent the ventricular zone of the posterior (medial tissue) and caudal parts of the anterior, basal or lateral (lateral tissue) amygdalar radial units, respectively. The radial histogenetic model of the pallial amygdala clarified that the ventricular zone of the amygdala lies more caudal to its respective derivatives, placing it in a region that shows strong *CoupTf2* expression (Kanatani et al., 2008, 2015; Tang et al., 2012; Ruiz-Reig et al., 2018; Garcia-Calero et al., 2020). None of the currently known progenitor regions of the AHi have been described to be dependent on the expression of *CoupTf2* for the generation or migration of their cells, nonetheless a strong reduction of cells within the AHi in CoupTf2 mutants has been reported. Tang et al. (2012), under the impression that the BM was largely generated from the CGE, discussed that a synergistic interplay between pallial and subpallial cells might be necessary for correct development of the BLC (Tang et al., 2012). This was based upon the observation that not only the BM, which shows strong *CoupTf2* expression, but also the BLA and LA were disrupted in *CoupTf2* mutants. However, the radial unit model indicates that principal BM cells originate from the amygdalar pallial division (anterior and basal unit) instead of the subpallial CGE. A more likely interpretation is therefore that BM, BLA, and LA receive a subset of *CoupTf2* expressing cells, and thus the “*CoupTf2*+ CVP-like” progenitor region might also partly overlap with the progenitor zone of the anterior, basal, and/or lateral radial unit, similar to previous findings in chicken and sauropsids as reviewed in Medina et al. (2021). While only the MA was identified as a derivative of the CVP, Ruiz-Reig et al. (2018) based themselves upon the expression of a single marker gene (*Ebf3*) to identify CVP-derivatives. It’s possible that they identified a subpopulation of a *CoupTf2*-positive caudal pallial amygdalar field. A reexamination of the *CoupTf2*, *Dbx1*, and *Emx1* fields with radial sections or lightsheet microscopy could reveal additional links between nuclei.

While the spatial regulation of amygdalar cell types might seem evident from this review, much less is understood about its temporal regulation. Nonetheless, a finely tuned temporal regulation is equally important, since the subpallial progenitor regions also provide cells for other brain nuclei. In general, specification of interneuron subtype seems strongly dependent on their time of birth (see Bandler et al., 2017). *Sst*-expressing interneurons for the amygdala might be generated simultaneously with those destined for the cortex, as seems evident from their migratory route, and thus spatial rather than temporal factors likely influence their preferred trajectory toward the amygdala. In contrast, subpallial amygdalar interneurons from the central nucleus (origin principal cells LGE) seem to be generated at earlier timepoints (CA: ∼E10.5) than interneurons from other LGE-derived structures [striatal (E11-E16) (Marìn et al., 2000), olfactory (E13.5-15.5) (Fuentealba et al., 2015)]. Likewise, *Emx1*-lineage cells from the BLC (pallial) were found to be generated earlier (E9.5-E11.5) than those in the Striatum (E11.5-E15.5) (Cocas et al., 2009). While the temporal regulation of other amygdalar cell types is currently not well-understood, it is likely that similar differences in their time of birth might specify cells to become amygdaloid. The importance of epigenetic regulation of cell fate has recently emerged, and this might provide an additional way that amygdalar fate is regulated in embryonic progenitor zones (reviewed in Nord et al., 2015). It is clear that further research is necessary to completely understand what factors drive cells to an amygdalar fate, especially since defects in fate specification and apoptosis might cause some of the aforementioned phenotypes observed in LOF models (see e.g., *Tshz1*).

The molecular mechanisms that control the migration and positioning of cells within the amygdala remain largely unknown. Expression of *CoupTf2*, *via* its regulation of *Nrp1/Nrp2* expression, might be a common mechanism that interneurons utilize to avoid the striatal area and to drive cells toward the caudal pole (PAS, CMS) (Yozu et al., 2005; Kanatani et al., 2008, 2015; Tang et al., 2012). The migratory route toward the BM identified by Tang et al. (2012), interpreted by us to be emerging from the pallial amygdalar field, is also dependent on *CoupTf2* expression. Similarly, cells within the “CVP,” a presumed caudal part of the pallial amygdalar field, strongly express *CoupTf2* and might thus likewise be dependent on this gene for their migration (Ruiz-Reig et al., 2018). While repellent *Nrp1/Nrp2* can explain the avoidance of the striatal area, the guidance cues acting downstream from *CoupTf2* that drive these cells caudally remain unknown. Peculiarly, a subset of identified migratory routes seem to be adjacent or partly overlap (Dg SvSpM, POA, CMS, HyA, best seen on horizontal schemes e.g., Figures 7D, 8B, 11B) at the caudomedial telencephalon (Yozu et al., 2005; Kanatani et al., 2015; Puelles et al., 2016b; Touzot et al., 2016; Garcia-Calero et al., 2021). This implicates that similar molecular cues might confine migrating cells to these streams, and subsequently guide them toward the amygdala. The factors that drive cvMGE/Dg-derived interneurons and SPV-derived neurons toward this route are also poorly understood. Likewise, studies on how *Otp* seems to non-autonomously affect migration of some *Sim1+/Otp-* cells destined for the BMA (and perhaps CA) could shed light on the molecular factors that guide their collective migration through the HyA. As the existence of a separate amygdalar pallial field has only recently been elucidated, the molecular mechanisms involved in outside-in aggregation of pallial amygdalar nuclei remain largely unknown. Nonetheless, based on available data, it seems mostly independent from *Reelin* and *Cdk5*, and might require *Lis* and *Dcx* (Carney et al., 2006; Bai et al., 2008; Boyle et al., 2011).

The majority of migration studies focused on transcription factors and guidance cues. However, cellular migration is also strongly affected by cell-extracellular matrix interactions and direct cell-cell communication. One superfamily of adhesion molecules, the Cadherins, show an extensive mosaic expression pattern in the rodent postnatal amygdala, and even seem to delineate several amygdalar nuclei (Hertel et al., 2012). Misexpression of Protocadherins has previously been linked to migratory, axon guidance and synaptogenesis defects [an overview of the involvement of protocadherin in several signaling pathways is given in Pancho et al. (2020)]. While the role of (proto)cadherins in axon outgrowth and synaptogenesis has been studied in the amygdala, a putative effect on migration has not been described (Hayashi et al., 2014; Chang et al., 2017; Schoch et al., 2017). Likewise, evidence that other adhesion molecules influence the migration of amygdalar neurons is currently lacking, since most studies involving adhesion molecules seem to focus on neuronal connectivity. In addition to studies that further characterize the various origins of amygdalar cells (see above), a better understanding of the synergy between guidance cues, transcription factors ánd cell-cell communication is crucial to unravel the specification of cellular fate and migration of amygdalar cells.

The amygdala has been linked to a plethora of neurodevelopmental and neuropsychiatric disorders, the majority of which show phenotypes related to anxiety (fear) and social behavior (monitoring of emotional stimuli). A reduced number, misplacement or misspecification of cells within the amygdala fear circuit could directly cause altered anxiety or fear thresholds. Although axon guidance and synaptogenesis are likewise critical in this regard, these processes are in turn dependent on the presence of the correct cell type at the correct time during development. To better treat neurodevelopmental disorders, we need to first comprehend what processes occur in what timeline in a normal brain. Similarly, a better understanding of normal amygdalar development can directly help us understand the changes and their effect that occur during and after traumatic events. As groundbreaking discoveries frequently emerge and many questions surrounding the amygdala remain unanswered, developmental research surrounding the amygdala remains highly relevant today.

## Author Contributions

TA wrote all the text and created all the figures. ES thoroughly reviewed the text and gave suggestions for edits. Both authors contributed to the article and approved the submitted version.

## Conflict of Interest

The authors declare that the research was conducted in the absence of any commercial or financial relationships that could be construed as a potential conflict of interest.

## Publisher’s Note

All claims expressed in this article are solely those of the authors and do not necessarily represent those of their affiliated organizations, or those of the publisher, the editors and the reviewers. Any product that may be evaluated in this article, or claim that may be made by its manufacturer, is not guaranteed or endorsed by the publisher.
